# p53 isoform expression promotes a stemness phenotype and inhibits doxorubicin sensitivity in breast cancer

**DOI:** 10.1038/s41419-023-06031-4

**Published:** 2023-08-08

**Authors:** Luiza Steffens Reinhardt, Kira Groen, Xiajie Zhang, Brianna C. Morten, Anna Wawruszak, Kelly A. Avery-Kiejda

**Affiliations:** 1 School of Biomedical Sciences and Pharmacy, College of Health, Medicine and Wellbeing, The University of Newcastle, Callaghan, NSW Australia; 2 Hunter Medical Research Institute, New Lambton, NSW Australia; 3 Cancer Detection & Therapy Research Program, Hunter Medical Research Institute, New Lambton, NSW Australia; 4 Department of Biochemistry and Molecular Biology, Medical University of Lublin, Lublin, Poland

**Keywords:** Breast cancer, Preclinical research, Experimental models of disease, Cancer stem cells, Cellular imaging

## Abstract

In breast cancer, dysregulated *TP53* expression signatures are a better predictor of chemotherapy response and survival outcomes than *TP53* mutations. Our previous studies have shown that high levels of Δ40p53 are associated with worse disease-free survival and disruption of p53-induced DNA damage response in breast cancers. Here, we further investigated the in vitro and in vivo implications of Δ40p53 expression in breast cancer. We have shown that genes associated with cell differentiation are downregulated while those associated with stem cell regulation are upregulated in invasive ductal carcinomas expressing high levels of Δ40p53. In contrast to p53, endogenous ∆40p53 co-localised with the stem cell markers Sox2, Oct4, and Nanog in MCF-7 and ZR75-1 cell lines. ∆40p53 and Sox2 co-localisation was also detected in breast cancer specimens. Further, in cells expressing a high ∆40p53:p53 ratio, increased expression of stem cell markers, greater mammosphere and colony formation capacities, and downregulation of *miR-145* and *miR-200* (p53-target microRNAs that repress stemness) were observed compared to the control subline. In vivo, a high ∆40p53:p53 ratio led to increased tumour growth, Ki67 and Sox2 expression, and blood microvessel areas in the vehicle-treated mice. High expression of ∆40p53 also reduced tumour sensitivity to doxorubicin compared to control tumours. Enhanced therapeutic efficacy of doxorubicin was observed when transiently targeting Δ40p53 or when treating cells with OTSSP167 with concomitant chemotherapy. Taken together, high Δ40p53 levels induce tumour growth and may promote chemoresistance by inducing a stemness phenotype in breast cancer; thus, targeting Δ40p53 in tumours that have a high Δ40p53:p53 ratio could enhance the efficacy of standard-of-care therapies such as doxorubicin.

## Introduction

Nearly all deaths from breast cancer are a result of resistance to treatment and the subsequent development of metastases [1]. Understanding the mechanisms that contribute to deregulated p53 activities may reveal novel avenues for increasing the sensitivity to commonly used therapies in breast cancer.

In breast cancer, dysregulated p53 expression signatures are a better predictor of outcome and chemotherapy response than *TP53* mutation [2, 3], suggesting that alternative molecular mechanisms may compromise p53 function. In recent years, the complexity of p53 signalling has become increasingly apparent with the discovery that p53 is expressed as 12 isoforms whose expression is associated with clinical features and outcomes of human cancers [4–11], and whose cellular activities can modulate cell fate decisions [5, 10, 12–19]. This suggests that the imbalanced expression levels of p53 isoforms to p53 are important in cancer prognostication and may affect the response to therapies known to activate the p53 pathway, such as DNA-damaging chemotherapies.

We have demonstrated that high levels of the Δ40p53 isoform supress cellular mobility and proliferation [18], however, following doxorubicin (DOX), a high Δ40p53:p53 ratio impairs the canonical p53 DNA damage response [20]. In support of this, a study from our group has shown that Δ40p53 is potentially associated with cisplatin response in melanoma [21]. Moreover, this variant is capable of controlling p53 folding, oligomerisation, and post-translational modifications (PTMs) in a DOX-dependent fashion [22].

Δ40p53 expression has been shown to play a fundamental role in maintaining a highly proliferative and pluripotent state and inhibiting a more differentiated state in mouse embryonic cells [23]. In a cancer context, increased levels of Δ40p53 were detected in glioblastoma, but not in the cerebral cortex, which is predominantly composed of differentiated cells, moreover, its expression profile in glioblastoma xenografts resembled highly proliferative and undifferentiated stem cells [24]. Our previous studies have shown that, at the RNA level, a high Δ40p53:p53 ratio is significantly associated with worse disease-free survival in breast cancer [6, 11], and that at the protein level, Δ40p53 is expressed in a subpopulation of cells within the microenvironment of invasive ductal carcinomas (IDCs) [25] and breast cancer cell lines [20]. Thus, endogenously expressed Δ40p53 may adversely affect survival by promoting treatment resistance and recurrence, traits typically associated with cancer stem cells (CSCs). Hence, in this study, we aimed to uncover the relationship between Δ40p53, CSC regulation, and treatment outcomes in breast cancers.

## Results

### Differential gene expression in IDCs with high versus low Δ40p53

**Fig. 1:**
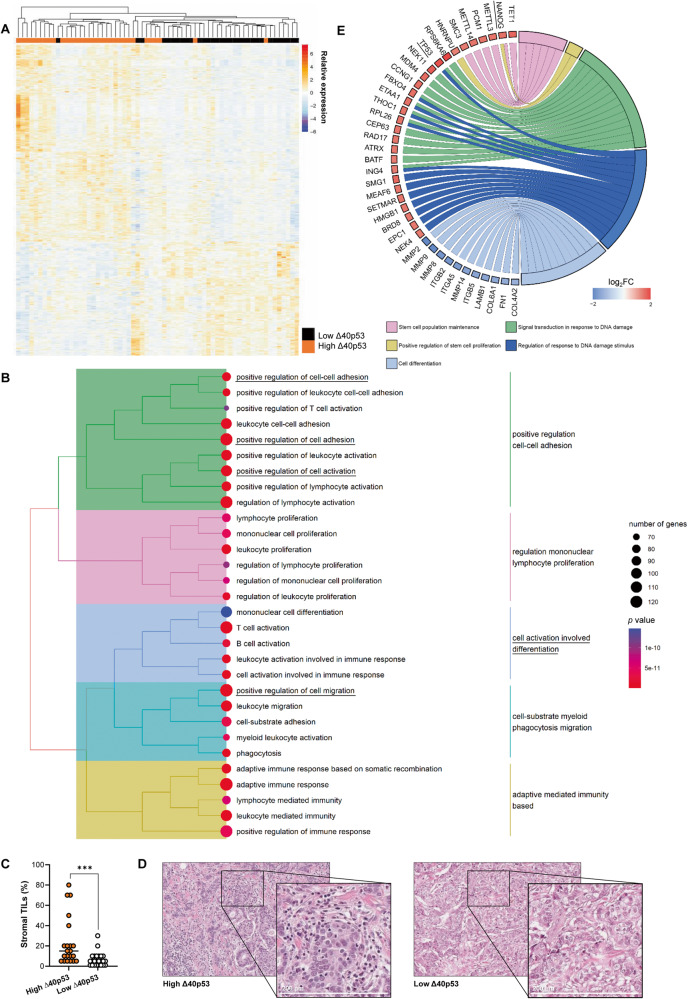
High Δ40p53 expression is associated with altered gene expression in IDCs. **A** Heatmap of 64 IDCs divided into high (*n* = 32) and low (*n* = 32) Δ40p53 expression as determined by RT-qPCR using median Δ40p53 expression as the cut-off [18]. The relative expressions (row z-scores) were computed for genes that are significantly differentially expressed (FDR corrected *p*-value < 0.05). Similarity in the expression between genes and between samples (branches on top) was measured using Euclidean hierarchical clustering. Relative expression with a red colour indicates higher expression of a gene and blue colour indicates lower expression of a gene. **B** Tree plot of top 30 gene ontology (GO) terms enriched in downregulated genes in high Δ40p53 versus low samples. Relevant terms for the study are underlined. **C** Percentage of TILs quantified in 47 IDCs divided into high and low Δ40p53 expression. **D** Representative H&E images of high Δ40p53 and low Δ40p53-expressing IDC samples. **E** Functional annotations of differentially expressed genes that are upregulated or downregulated in high Δ40p53 versus low samples. Blue fields indicate genes that exhibit decreased expression and red fields indicate genes that exhibit increased expression. Statistical analysis was carried out using an unpaired Mann–Whitney test (**C**). Results were considered significant at *p* < 0.05; ***p < 0.001.

Data from HumanGene1.0 Arrays on 64 breast tumours comprising 11 Grade 1, 6 Grade 2, and 47 Grade 3 IDCs (GSE accession no. 61725; Supplementary Table 1) were reanalysed to combine oestrogen-receptor negative and positive IDCs and samples were classified based on high or low Δ40p53 RNA expression with the median expression used as the cut-off (*n* = 32/group), which was previously evaluated by our research group using RT-qPCR [6, 18] (Fig. 1A). Hierarchical clustering was performed on transcripts found to be differentially expressed (*p* < 0.05) in high (orange) versus low (black) Δ40p53-expressing IDCs. Similarity between samples (branches on top) point toward a pattern of expression based on Δ40p53 levels (Fig. 1A) and 2257 genes (mapped IDs: 2162) were significantly downregulated whereas 1616 (mapped IDs: 1149) were upregulated when Δ40p53 was highly expressed (*p* < 0.05; log_2_(fold change) >|1|; 5% FDR; Supplementary Tables 2 and 3). GO analysis of transcriptomic data revealed that the majority of GO terms enriched in downregulated genes are associated with immune responses (Fig. 1B). Given that the tissue samples used for this analysis contain immune cells such as lymphocytes and plasma cells [25], H&E sections were assessed for tumour-infiltrating lymphocytes (TILs) within the breast tumours microenvironment. An increased percentage of TILs was observed in samples with high Δ40p53 expression when compared to those with low Δ40p53 expression (Fig. 1C, D), possibly explaining the difference in immune pathway enrichment between the groups. In support of this, Δ40p53 co-localised with a plasma cell marker (CD38) [26] in formalin-fixed paraffin-embedded (FFPE) slides of three ER (oestrogen receptor)+/PR (progesterone receptor)+/Her2 (human epidermal growth factor receptor 2)- IDCs (Supplementary Fig. 1), suggesting that this isoform is expressed in immune cells within the tumour microenvironment. Nevertheless, Δ40p53 was also expressed in a subpopulation tumour cells (Supplementary Fig. 1).

In addition to immune-related regulation, GO terms associated with positive regulation of cell adhesion and cell activation involved in differentiation pathways (Fig. 1B), and several other pathways including terms associated with regulation of cell shape and apoptotic signalling were downregulated in cells where Δ40p53 was highly expressed (Supplementary Table 4). GO analysis for upregulated genes in cells expressing high Δ40p53 showed enrichment of terms related to cilium assembly chain movement, post-transcriptional gene silencing, and regulation of mRNA processing (Supplementary Fig. 2, Supplementary Table 5). The analysis of specific pathways in high versus low Δ40p53 samples demonstrated upregulation of signal transduction in response to DNA damage and regulation of response to DNA damage stimulus-related genes, including *TP53* (Fig. 1E). In addition, genes linked to stem cell maintenance and proliferation, including a core stemness transcription factor, *NANOG*, were upregulated in high versus low Δ40p53 IDCs, whereas genes related to cell differentiation were downregulated (Fig. 1E).

### High levels of Δ40p53 induce stem cell marker expression

Given that several downregulated genes in high versus low Δ40p53 samples were enriched in GO terms associated with cell differentiation and adhesion regulation and that Δ40p53 is expressed in a small percentage of cells within IDCs [25] and breast cancer cell lines [20], we investigated if Δ40p53 expression was related to breast cancer cell de-differentiation and CSCs.

**Fig. 2:**
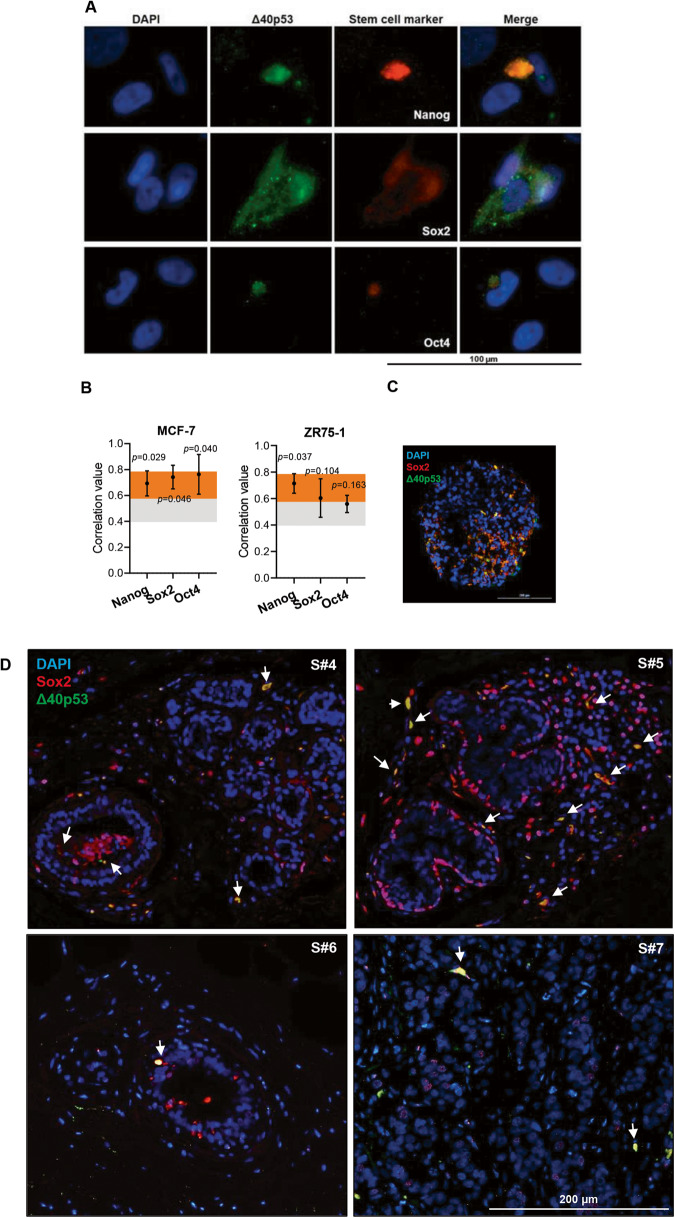
Δ40p53 co-localises with stem cell markers. **A** Immunofluorescence images of Δ40p53 and Nanog, Sox2, or Oct4 staining in the MCF-7 cell line. KJC40 (5 μg/ml) and stem cell markers Nanog (20 μg/ml), Sox2 (5 μg/ml), and Oct4 (2 μg/ml) primary antibodies were used and cell nuclei were stained with DAPI. **B** Correlation values of the co-localisation of Δ40p53 and Nanog, Sox2, or Oct4 immunofluorescent staining in MCF-7 and ZR75-1 cells. Orange and grey shaded areas indicate strong or moderate correlation values, respectively. **C** Immunofluorescence images of Δ40p53 and Sox2 staining in a ZR75-1 cell spheroid and (**D**) in breast cancer specimens (*n* = 5: S#1–5). Arrows indicate regions of co-localisation. KJC40 (5 μg/ml (**C**) and 8 μg/ml (**D**)) and Sox2 (5 μg/ml) primary antibodies were used and cell nuclei were stained with DAPI. For single staining of fluorescence channels of merged (**C**) and (**D**) figures, see Supplementary Fig. 4. Analyses were carried out using ImageJ (Coloc 2) and Spearman’s rank correlation was used for the co-localisation analyses. For co-localisation analysis in MCF-7 and ZR75-1 cell lines, four images were evaluated per well (~30 cells were evaluated per triplicate). For co-localisation analysis in IDCs slides, ten microscope fields were collected per slide. Results were considered significant at *p* < 0.05. S: specimen.

To evaluate if Δ40p53 expression was associated with stem cell marker expression, immunofluorescence was performed on MCF-7 cells and ZR75-1 breast cancer cells. Δ40p53 or p53 antibodies were co-stained with antibodies that detect transcription factors that regulate stem cells (Nanog, Sox2, or Oct4). Δ40p53, which we have previously shown to localise in both the nucleus and cytoplasm in these cells [20], co-localised with Nanog, Sox2, and Oct4 (which are localised mainly in the nucleoplasm) in both breast cancer cell lines (Fig. 2A, B) (*p* < 0.05 for Nanog, Sox2, and Oct4 in MCF-7 and Nanog in ZR75-1). The co-localisation of Δ40p53 and Sox2 was further confirmed in 3D ZR75-1 cell spheroids (Fig. 2C) and in five (S#4–7: ER+/PR+/Her2- and S#8: ER-/PR-/Her2+) IDCs (correlation value: 0.52; *p* < 0.05) (Fig. 2D), indicating that Δ40p53 is expressed in more pluripotent breast cancer cells. In contrast, p53 did not co-localise with either of the three stem cell markers (Supplementary Fig. 3), suggesting that these isoforms play divergent roles in regulating pluripotency and differentiation.

**Fig. 3:**
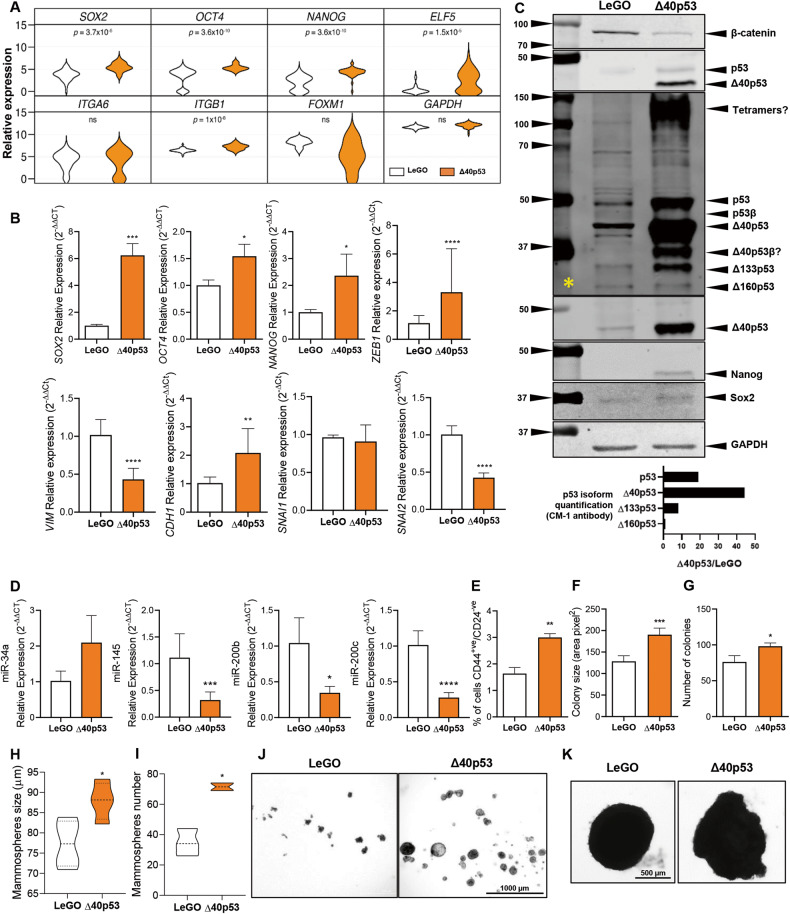
High Δ40p53 expression is associated with increased stem cell marker expression. **A** Relative expression of *SOX2, OCT4, NANOG, ELF5, ITGA6, ITGB1*, *FOXM1*, and *GAPDH* of single cells in MCF-7-LeGO and MCF-7-Δ40p53 sublines quantified by single-cell RT-qPCR. Data shown represent one independent experiment. The violin plots indicate the cell population distribution. **B** mRNA expression levels of *SOX2*, *OCT4*, *NANOG, ZEB1, VIM, CDH1, SNAI1*, and *SNAI2* in MCF-7-LeGO and MCF-7-Δ40p53 sublines quantified by bulk RT-qPCR. Data shown represent three independent experiments of three technical replicates. **C** Top: Representative immunoblotting of MCF-7-LeGO and MCF-7-Δ40p53 cell extracts (40 µg). β-catenin (1 μg/ml), CM-1 (1 μg/ml), KJC40 (2.5 μg/ml), Nanog, (1 μg/ml), Sox2 (1 μg/ml), and GAPDH (1 μg/ml; loading control) primary antibodies were used. * indicates an immunoblot probed for CM-1 when high contrast is applied to the membrane imaging. Bottom: quantification of p53 isoform expression in MCF-7-Δ40p53 subline normalised to p53 isoform expression in MCF-7-LeGO subline. **D** Relative expression of *miR-34a*, *miR-145*, *miR-200b*, and *miR-200c* in MCF-7-LeGO and MCF-7-Δ40p53 sublines. Data shown represent three independent experiments of three technical replicates. **E** Percentage of CD44 positive and CD24 negative cells (quantified by Flow cytometry), **F** colony size, **G** number of colonies, **H** mammosphere size (>60 µM), and **I** mammosphere number in MCF-7-LeGO and MCF-7-Δ40p53 sublines. Data shown represent three independent experiments of three technical replicates. **J** Representative images of mammosphere formation assay in MCF-7-LeGO and MCF-7-Δ40p53 sublines. **K** Representative images of MCF-7-LeGO and MCF-7-Δ40p53 cell spheroids. Results are shown as the mean ± SD (**A**–**E**, **H**, **I**) or mean ± SEM (**F**, **G**). Statistical analyses were carried out using an unpaired t-test. Results were considered significant at *p* < 0.05; *p < 0.05, **p < 0.01, ***p < 0.001, ****p < 0.0001.

p53 maintains the balance between self-renewal and differentiation and is a known indirect transcriptional repressor of stem cell markers and an inhibitor of the epithelial-mesenchymal transition (EMT) signalling, which leads to the acquisition of stem-like properties by epithelial cells and generation of CSCs [27]. Given that p53 inactivation disrupts this balance and promotes pluripotency and somatic cell reprogramming, we next explored the association of Δ40p53 with cell pluripotency by using MCF-7 sublines in which Δ40p53 (α, β, and γ) has been stably knocked down (-shΔ40p53) or overexpressed (Δ40p53α, which will be referred to as Δ40p53 for simplicity) [18]. In the Δ40p53 overexpression cells, we have previously observed increased expression and stability of p53, in spite of that, these cells show a high Δ40p53:p53 ratio [20]. An empty vector (LeGO), a non-targeting control shRNA (-shNT), and a full-length transactivation p53 knockdown (-shp53) sublines were also used [18]. The expression of stem cell and EMT markers was examined by single-cell RT-qPCR (Fig. 3A) and bulk RT-qPCR (Fig. 3B).

At the single-cell level, the expression of *SOX2*, *OCT4*, *NANOG*, *ELF5* (inhibits the transcription of *SNAI2* and represses EMT [28] and proliferation of breast cancer cells [29]), and *ITGB1* (promotes cell motility and contributes to the EMT [30]) were significantly increased in Δ40p53 cells compared to LeGO cells (Fig. 3A). The upregulation of *SOX2*, *OCT4,* and *NANOG* was confirmed by bulk RT-qPCR (Fig. 3B), immunofluorescence (Supplementary Fig. 5), and western blotting (for Nanog and Sox2; Fig. 3C). Bulk RT-qPCR revealed increased expression of *ZEB1* (regulator of cell plasticity and DNA damage response [31]) and *CDH1* (E-cadherin), but a decreased expression of *VIM* (vimentin), *SNAI2* (repress E-cadherin and promotes EMT [32]) (Fig. 3B), and β-catenin (Fig. 3C) in Δ40p53 cells compared to LeGO cells. Of note, in Δ40p53 cells, it was observed increased expression of p53 and Δ133p53 by 20 and 10-fold, respectively (Fig. 3C).

Decreased expression of *SOX2*, *OCT4*, *NANOG,* and *ZEB1*, and increased expression of *SNAI1* (promotes EMT [32]) were detected in -shΔ40p53 cells, whereas increased expression of *SOX2*, *VIM*, and *SNAI2*, and decreased expression of *CDH1* were detected in -shp53 cells (Supplementary Fig. 6). These results underpin that p53 and Δ40p53 may play different roles in modulating the levels of stem cell and EMT regulators in breast cancer cells and that high levels of Δ40p53 may promote pluripotency and stem cell maintenance but not de-differentiation. On the other hand, Δ40p53 knockdown may contribute to an increased EMT-related transcriptional programme and cell motility, supporting our previous findings [18] and contrasting p53 knockdown, which may result in cell pluripotency and EMT.

One of the described mechanisms by which p53 regulates the expression of stem cell and EMT markers is by promoting the expression of microRNAs (miRNAs), which then control the expression of those genes [27], hence, to further understand the mechanisms behind the upregulation of pluripotency markers in Δ40p53 cells, the expression p53-targetting miRNAs was evaluated. *miR-145* (represses Sox2 and Oct4 [33, 34]), *miR-200b* (represses Zeb1 [35]), and *miR-200c* (represses Zeb1 [36]) were found downregulated in Δ40p53 cells when compared to LeGO cells (Fig. 3D), suggesting miR-mediated translational inhibition is a possible mechanism for the increased levels of Sox2, Oct4, Nanog, and Zeb1 in Δ40p53 cells. No significant differences were detected in *miR-34a* (represses Oct4, EMT and stemness [37]) (Fig. 3D).

### High levels of Δ40p53 induce stemness phenotype

We next evaluated the effect of high Δ40p53 levels on cell phenotype by evaluating CD44 and CD24 levels and mammosphere and colony formation capacities. Δ40p53 cells presented an increased percentage of CD44^+ve^/CD24^-ve^ cells (Fig. 3E), size and number of colonies (Fig. 3F, G), and mammosphere size and number compared to LeGO cells (Fig. 3H–J). Interestingly, cell spheroids generated with Δ40p53 cells presented a loose morphology at 14 days in culture (Fig. 3K), indicating that Δ40p53 expression may induce a shift in cell morphology from a paved stone epithelial appearance (such as LeGO cell spheroids) to an irregular shape. In order to evaluate if the morphological differences between LeGO and Δ40p53 spheroids were due to cell polarity, the orientation of the Golgi apparatus was evaluated using GM130 (Golgi protein) staining [38]. Compared to LeGO spheroids, cells from Δ40p53 spheroids presented diffuse staining with varying orientation, similar to MCF-7 2D cultures [39], with many cells having staining present on the apical surface and others with staining on the basal and lateral sides. LeGO cells tend to present more consistent staining with small punctate areas toward the edge plasma membrane; however, diffuse staining was also detected in these cells (Supplementary Fig. 7; arrows indicate the direction of the polarity determined by the position of the GM130 staining; the orientation of cells with diffuse staining was not possible to determine).

These findings support the microarray results (Fig. 1) where genes related to cell shape regulation and cell polarity were downregulated in high versus low Δ40p53 cases (Supplementary Tables 2–5). Overall, these results indicate that increased levels of Δ40p53 may affect cell morphology and promote a stemness phenotype in breast cancers. Yet, additional studies are needed to clarify how Δ40p53 affects cell polarity and if this is associated with the differentiation state of these cells.

### Δ40p53 knockdown affects breast cell morphogenesis

**Fig. 4:**
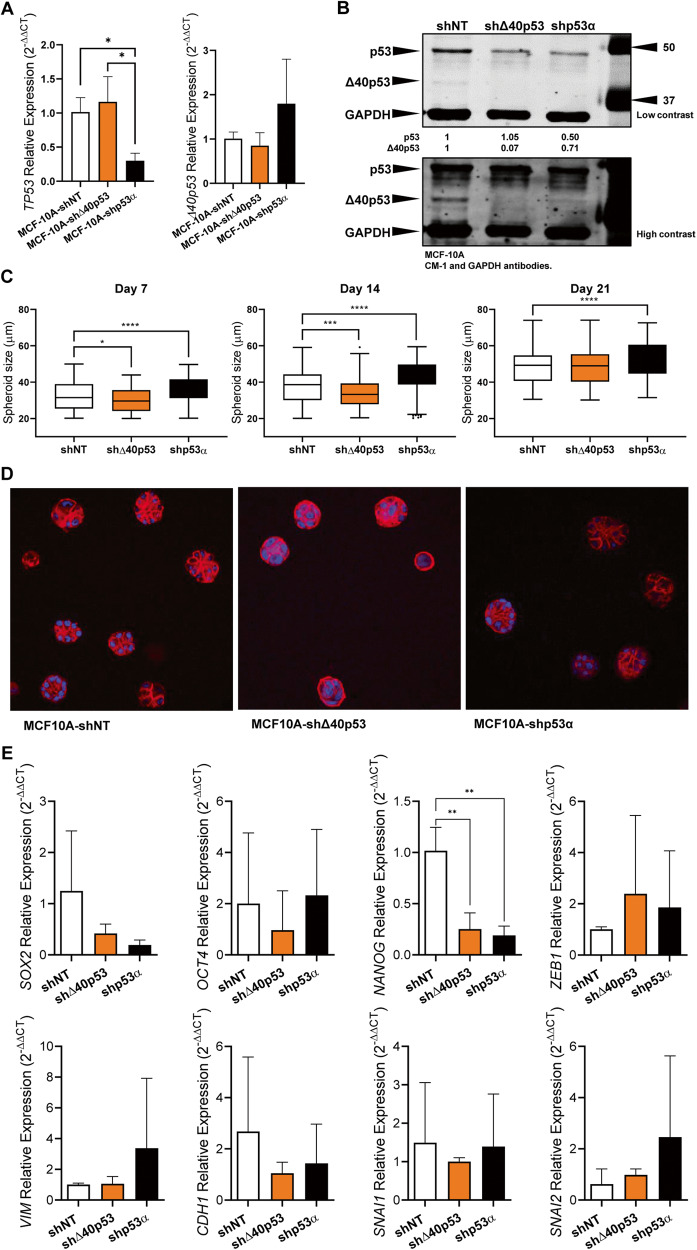
Altered Δ40p53 expression affects breast cell morphogenesis. **A** mRNA expression levels of *TP53* and *Δ40p53* in MCF-10A-shNT, MCF-10A-Δ40p53, and MCF-10A-shp53 cell sublines. Data shown represent three independent experiments of three technical replicates. **B** Representative immunoblotting of MCF-10A cell subline extracts (40 µg). CM-1 (1 μg/ml) and GAPDH (1 μg/ml; loading control) primary antibodies were used. **C** Acini size quantification in MCF-10A-shNT, MCF-10A-Δ40p53, and MCF-10A-shp53 sublines. Data shown represent three independent experiments of three technical replicates. **D** Representative images of phalloidin staining on MCF-10A-shNT, MCF-10A-Δ40p53, and MCF-10A-shp53 acini on day 21. **E** mRNA levels of *SOX2*, *OCT4*, *NANOG, ZEB1, VIM, CDH1, SNAI1,* and *SNAI2* in MCF-10A-shNT, MCF-10A-Δ40p53 and MCF-10A-shp53 sublines. Data shown represent three independent experiments of three technical replicates. Results are shown as the mean ± SD. Statistical analyses were carried out using one-way ANOVA followed by Dunnett’s post-test. Results were considered significant at *p* < 0.05; *p < 0.05, **p < 0.01, ***p < 0.001, ****p < 0.0001.

In order to elucidate if the effects of Δ40p53 knockdown were specific to tumour cells, acini formation assays were performed in normal human mammary epithelial cells MCF-10A in which Δ40p53 or p53 were stably knocked down (MCF-10A-shΔ40p53 and MCF-10A-shp53, respectively; Fig. 4A, B). This assay allows us to study the well-defined programme of proliferation and differentiation during the formation of polarised acinar-like structures that recapitulate several aspects of mammary architecture. After 7 and 14 days in three-dimensional culture, MCF-10A-shΔ40p53 spheres were smaller than MCF-10A-shNT spheres, whereas MCF-10A-shp53 spheres were larger even though p53 was only partially knocked down (Fig. 4C). Following 21 days in culture, MCF-10A-shp53 spheres presented a larger size compared to MCF-10A-shNT spheres (Fig. 4C), most likely due to reduced apoptosis induction within the inner cells (important phase in acini formation [40]). Acini from MCF-10A-shNT cells presented a spherical structure with a smooth outer edge and the individual cells at that edge appeared to be of uniform size and evenly spaced (Fig. 4D). The knockdown of p53 generated structures with an acinar appearance but these acini were larger than those for the MCF-10A-shNT cells and were more irregular in shape (Fig. 4D). Δ40p53 knockdown led to smaller and rounder structures, with smooth outer edges, and disorganised individual cells, indicating less mature acini (Fig. 4D). These results suggest that the knockdown of Δ40p53 may interfere with breast cell morphogenesis and organoid developmental outcome. With the exception of *NANOG*, which was found to be downregulated in MCF-10A-shΔ40p53 and MCF-10A-shp53 cells compared to MCF-10A-shNT cells (Fig. 4E), no other significant differences were observed in stem cell and EMT marker expression between the MCF-10A sublines (Fig. 4E), however, the gene expression analysis was performed using two-dimensional cultures, which may not represent the transcriptional regulation of acini.

Not surprisingly, the function of Δ40p53 may differ between normal and cancerous cells. In MCF-10A cells, the lack of Δ40p53 may affect morphogenesis, but in MCF-7 cells, its knockdown may induce increased cell migration [18].

### Altered levels of Δ40p53 affect sensitivity to DOX treatment

**Fig. 5:**
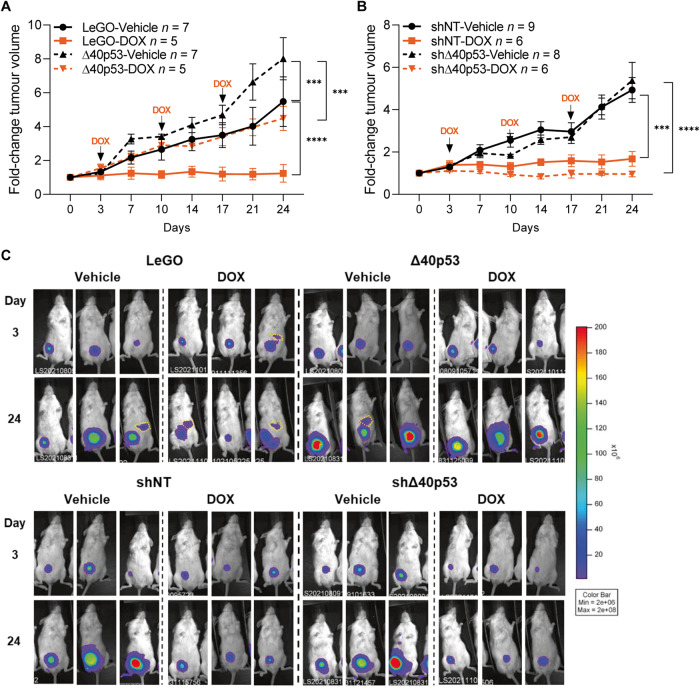
High levels of Δ40p53 induce tumour growth and lead to decreased sensitivity to DOX in mouse xenografts. **A** Tumour volume normalised to tumour size on day 0 (prior treatment) of **A** MCF-7-LeGO (solid lines) and MCF-7-Δ40p53 (dashed lines) and **B** MCF-7-shNT (solid lines) and MCF-7-shΔ40p53 (dashed lines)-derived xenografts treated with saline (black lines) or DOX (orange lines). **C** Representative images of luminescent imaging of mice immediately after subcutaneous administration of luciferin. Yellow dashed circles demonstrate luciferin signal in spleens (see Supplementary Fig. 9C for details on mice spleens). Results are shown as the mean ± SD. Statistical analyses were carried out using two-way ANOVA followed by Tukey’s post-test. Results were considered significant at *p* < 0.05; ***p < 0.001, ****p < 0.0001.

Given that high Δ40p53 levels may contribute to stem cell population maintenance, which may contribute to treatment resistance and metastasis in cancer [41], we next assessed if altered levels of Δ40p53 could affect breast tumour growth and treatment responses in vivo. NGS mice were orthotopically injected with luciferase-labelled MCF-7-shNT, -shΔ40p53, -LeGO, or -Δ40p53 cells and treated with DOX for 3 weeks (Fig. 5). In vehicle-treated mice, increased tumour volume was detected in Δ40p53 tumours compared to LeGO (*p* < 0.001) (Fig. 5A). In DOX-treated mice, LeGO tumours did not grow, whereas high levels of Δ40p53 significantly decreased tumour sensitivity to the treatment (DOX-treated tumours; Δ40p53 vs LeGO: *p* < 0.0001) (Fig. 5A). In the knockdown models, no differences were detected in tumour volume for -shNT and -shΔ40p53 in vehicle-treated mice, and both cell sublines responded to DOX treatment (vehicle vs DOX; -shNT: *p* < 0.001, -shΔ40p53: *p* < 0.0001) (Fig. 5B). Although not statistically significant, an increase in DOX sensitivity was observed in -shΔ40p53 tumours when compared to -shNT tumours in treated mice (Fig. 5B).

### Δ40p53 is positively correlated with Ki67 expression in breast tumours

**Fig. 6:**
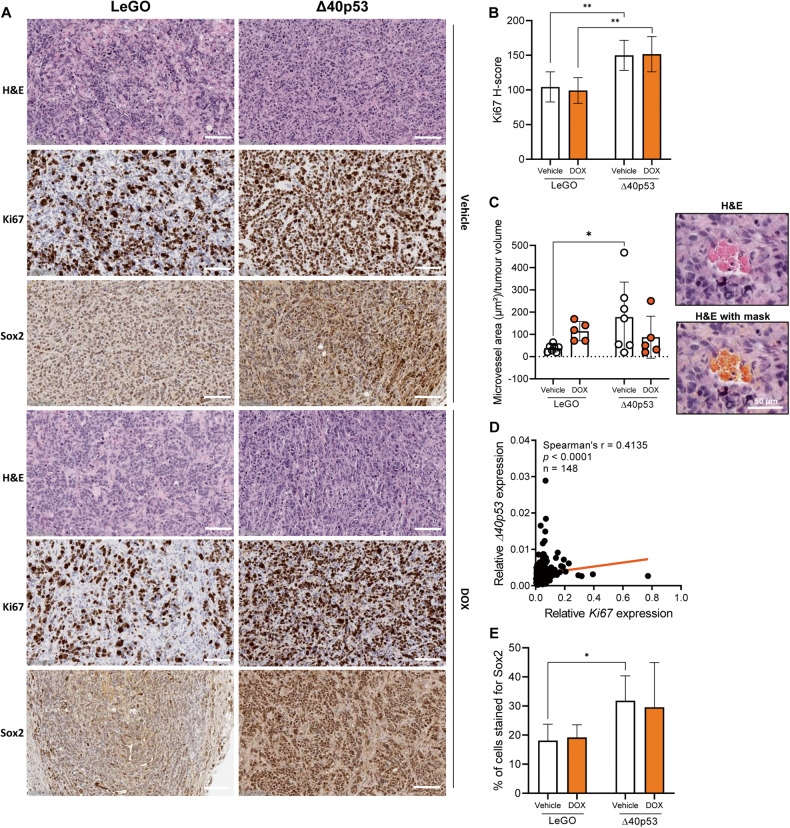
Xenograft tumours with high levels of Δ40p53 present increased expression of Ki67 and Sox2. **A** Representative images of H&E slides and slides stained for Ki67 or Sox2 in MCF-7-LeGO or MCF-7-Δ40p53 tumour xenografts treated with vehicle or DOX. Scale bars represent 100 µm. Ki67 (0.07 μg/ml) and Sox2 (2.5 μg/ml) primary antibodies were used. **B** H-score for Ki67 in MCF-7-LeGO or MCF-7-Δ40p53 tumour xenografts. **C** Correlation between *Δ40p53* and *Ki67* mRNA expression in a cohort of 148 breast cancers. **D** Percentage of cells strongly stained for Sox2 and **E** microvessel area normalised to tumour volume in MCF-7-LeGO or MCF-7-Δ40p53 tumour xenografts. Results are shown as the mean ± SD. Statistical analyses were carried out using two-way ANOVA followed by Sidak’s post-test. Results were considered significant at *p* < 0.05; *p < 0.05, **p < 0.01.

Given the increase in tumour volume when Δ40p53 is highly expressed, we evaluated the expression of the proliferation marker Ki67 by IHC in the xenograft tissues (Fig. 6A, B, Supplementary Fig. 8). Δ40p53 tumours presented increased Ki67 H-scores (Fig. 6B) and microvessel areas (Fig. 6C) compared to LeGO, supporting the tumour volume findings (Fig. 5A) and indicating that high levels of Δ40p53 induce tumour growth, which may be associated with tumour-related angiogenesis. In support of this, *Δ40p53* expression was positively correlated with *Ki67* expression (*r* = 0.4135, *p* < 0.0001) in 148 breast cancers (38 Grade 1, 38 Grade 2, and 72 Grade 3 IDCs [6]) (Fig. 6D), suggesting that high levels of Δ40p53 impact the proliferation of breast tumours. No differences in Ki67 expression were detected in the knockdown models (Supplementary Fig. 8).

In order to validate the in vitro findings supporting a role for Δ40p53 in CSC regulation (Figs. 2–4), we evaluated the nuclear expression of Sox2 in the xenograft tumours. Δ40p53 overexpression led to an increased percentage of cells strongly stained for Sox2 (Fig. 6E), supporting the hypothesis of enhanced stemness induced by increased Δ40p53 expression.

### Targeting Δ40p53 to decrease therapy resistance

**Fig. 7:**
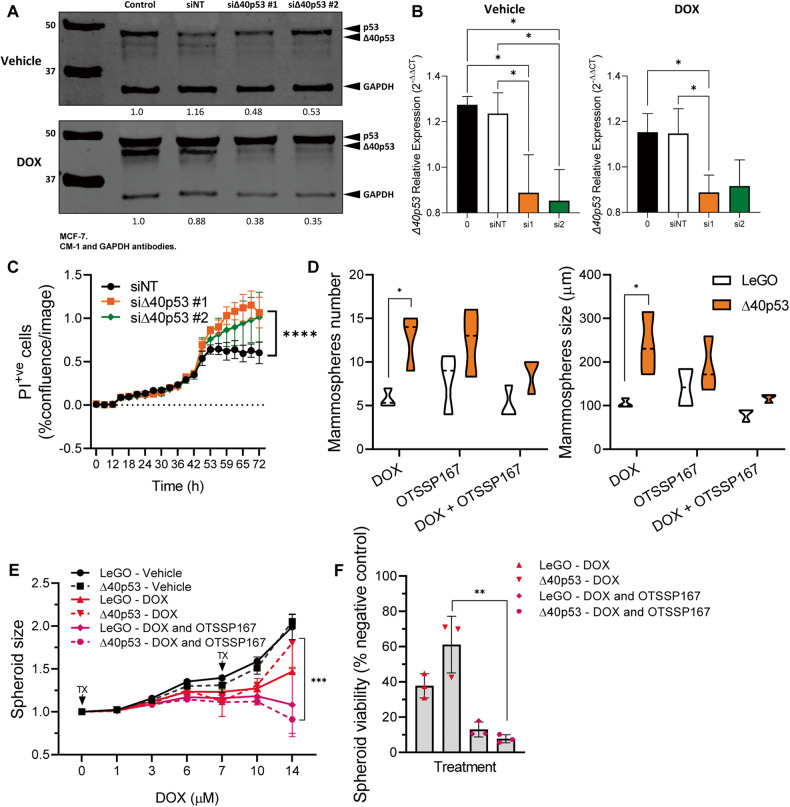
Targeting Δ40p53 increases DOX sensitivity. **A** Representative immunoblots of protein extracts (40 µg) of MCF-7 parental cells transiently transfected with siRNA targeting Δ40p53 (#1 and #2) or a non-targeting (NT) control and treated with vehicle or DOX for 24 h. CM-1 (1 μg/ml) and GAPDH (1 μg/ml; loading control) primary antibodies were used. **B** mRNA expression levels of *TP53* and *Δ40p53* in MCF-7 cells 24 h post-transfection and treatment with vehicle or DOX. Data shown represent three independent experiments of three technical replicates. **C** Propidium iodide (PI) positive cells normalised to confluence in MCF-7 cells transfected with siRNA targeting Δ40p53 (#1 and #2) or a NT control and treated with DOX. Data shown represent three independent experiments of three technical replicates. **D** Mammosphere size (>60 µM) and number in MCF-7-LeGO and MCF-7-Δ40p53 sublines treated with DOX, OTSSP167, or a combination of DOX and OTSSP167. Data shown represent three independent experiments of three technical replicates. **E** Spheroid size normalised to size prior treatment and **F** spheroid viability normalised to vehicle-treated spheroids in MCF-7-LeGO and MCF-7-Δ40p53 sublines treated with DOX, OTSSP167, or a combination of DOX and OTSSP167. Data shown represent three independent experiments of four technical replicates. Results are shown as the mean ± SD. Statistical analyses were carried out using one-way ANOVA followed by Tukey’s post-test (**B**, **F**) or two-way ANOVA followed by Sidak’s post-test (**C**–**E**). Results were considered significant at *p* < 0.05; *p < 0.05, **p < 0.01, ***p < 0.001, ****p < 0.0001.

The previous results indicate that high levels of Δ40p53 induce tumour growth, promote a stemness phenotype, and decrease DOX sensitivity of breast cancer cells, thus, we hypothesised that inhibiting Δ40p53 expression may improve treatment responses in cases where Δ40p53 is elevated (e.g., following DOX treatment [20]). Two siRNAs targeting intron 2 of *TP53* were used to knockdown Δ40p53 in MCF-7 cells (Fig. 7A, B). Cells were transfected and treated concomitantly with DOX. The knockdown of Δ40p53 led to a significant increase (*p* < 0.0001) in cell death when compared to the control (Fig. 7C).

Next, we targeted the Δ40p53-induced phenotype by targeting a serine/threonine-protein kinase, MELK, which is associated with DOX resistance [42], Sox2 upregulation [43], and *TP53*-mutant breast cancers [44] and was shown to be upregulated in Δ40p53 cells following DOX treatment [20]. LeGO and Δ40p53 cells were treated with a clinically relevant MELK inhibitor (ClinicalTrials.gov Identifier: NCT02926690), OTSSP167, alone or in combination with DOX (Fig. 7D–F). The results showed a decrease in mammosphere number and size (Fig. 7D) and in spheroid size and viability (Fig. 7E, F) in cells treated with the combined treatment compared to DOX alone, suggesting that inhibiting MELK may revert the resistance induced by high levels of Δ40p53 (and of MELK, which is also induced by DOX in Δ40p53 cells [20]). However, the treatment effects seen when treating cells with OTSSP167 may be also a result of off-target inhibition such as Aurora B, BUB1, and Haspin kinases, which needs to be further investigated [45].

## Discussion

It is becoming evident that canonical p53 function is linked to its isoforms [4, 8, 20, 46]; however, the knowledge on p53 isoform functions remains limited. Wild-type p53 maintains homeostasis between self-renewal and differentiation depending on the cellular and developmental state and prevents the de-differentiation and reprogramming of somatic cells to stem cells [27]. Yet, cancer stemness has emerged as a crucial oncogenic property of mutant *TP53* [27]. Little evidence has supported the role of p53 isoforms in driving a CSC phenotype [47] independently of *TP53* mutation status. Herein, we have shown that high Δ40p53 levels in breast cancer cells with wild-type *TP53* induce a stemness phenotype, increase tumour growth, and decrease sensitivity to DOX in vivo. In addition, a gene therapy approach of silencing Δ40p53 with concomitant chemotherapy could enhance the efficacy of DOX.

IDCs with high versus low Δ40p53 levels showed downregulation of genes associated with cell differentiation and upregulation of genes related to stem cell population maintenance (Fig. 1). Although caution must be taken when interpreting RNA levels of the p53 isoforms given their post-translation regulation [48, 49] and the lack of correlation between protein and RNA levels [25], these findings support the association of Δ40p53 expression with pluripotency [23, 24]. Increased expression of Δ40p53 was previously described in embryonic stem cells, where it inhibited the progression to a more differentiated state, supporting stem cell maintenance mediated by Nanog and the IGF-1 receptor [23]. Moreover, in glioblastoma xenografts, increased Δ40p53 expression was detected in cells that resembled highly proliferative and undifferentiated stem cells [24]. In this study, we have shown that Δ40p53 co-localises with stem cell markers in breast cancer cells (Fig. 2) and its overexpression led to an increase in pluripotency markers (*SOX2*, *OCT4*, *NANOG,* and *ZEB1*) and a stemness phenotype (Fig. 3).

As a CSC phenotype is extensively driven by epigenetic modulators, especially miRNAs [50], we evaluated the expression of p53-target miRNAs known to regulate the expression of the stem cell markers analysed in this study [33–37, 51–53]. The downregulation of *miR-145* and *miR-200* in Δ40p53 cells (Fig. 3D) is most likely caused by Δ40p53 loss-of-function since Δ40p53 may not undergo the same post-translational modifications as p53 [54], as a result, it may lack modifications that are required for p53 activation. For instance, in a context-specific manner, the physical interaction between TAD1 of p53 and other proteins enables some of p53 PTMs, which may drive miRNA expression (p53’s acetylation at the lysine^373^ by p300 [55]) or arouse transcriptional repression (p53’s phosphorylation at the serine^315^ residue by upstream kinases leads to p53 interaction with the *NANOG* promoter [56]). Similar to Δ40p53 cells, loss-of-function findings were observed in cells with inactivated *TP53* mutations [27, 36], suggesting that Δ40p53 functions are likely due to a loss of p53 functions and perhaps not a gain of independent roles [20]. Although we have not extensively proven the connection between an increased stemness phenotype and miRNA expression, we can assume that high Δ40p53 levels deregulate the p53-transcriptional regulation of these RNAs. Whether the modulation of miRNA expression is the only factor regulating the transcriptional differences between LeGO and Δ40p53 cells remains beyond the scope of this work, but it is highly unlikely to be the only mechanism given the complexity of the p53 pathway.

High levels of Δ40p53 also led to *CDH1* and *ELF5* upregulation and *VIM*, *SNAI2*, and β-catenin downregulation (Fig. 3), suggesting that even though Δ40p53 overexpression is associated with stemness and cell proliferation in vivo (Figs. 5, 6), it may not promote EMT or cell migration since no metastasis was observed in vivo. This supports our previous findings where Δ40p53 overexpression inhibited breast cancer cell migration similar to p53 [18]. Thus, the small percentage of IDCs, which express high levels of Δ40p53 [25] could drive tumour recurrence but may not induce metastasis. The expression of Sox2 has been previously found to be correlated with Ki67 index, larger tumours, and higher grade in IDC [57]. Tumour xenografts with high levels of Δ40p53 also showed increased Sox2 and Ki67 expression when compared to LeGO tumours (Fig. 6), highlighting the self-renewal properties of cells with overexpressed Δ40p53 and underpinning the association of Δ40p53 with worse outcomes [6, 11]. In addition, one important hallmark of cancer is the process of formation of new blood vessels from existing vasculature [58]. Mutant p53 promotes tumour neo-angiogenesis through the induction of reactive oxygen species and Hif1-α, which induces the expression of the pro-angiogenic factor VEGFA [59]. Δ40p53 most likely impairs the angiogenesis-related functions of canonical p53 in a similar manner given the increased microvessel areas in Δ40p53 tumours (Fig. 6C).

Importantly, the combined upregulation of other p53 isoforms may contribute to the Δ40p53 cell phenotype (Fig. 3C). It is known that high levels of Δ40p53 may lead to the formation of misfolded-p53 aggregates [60] and stabilisation of p53 isoform expression due to escape of proteasomal degradation mediated by HDM2 [20, 61, 62]. This could account for the increased expression of p53 and Δ133p53 in Δ40p53 cells by 20 and 10-fold, respectively (Fig. 3C). In this context, the elevated expression of the Δ133p53 isoforms [47] may contribute to the promotion of CSC features and other biological effects (e.g., enhanced angiogenesis) observed in Δ40p53 cells. Hence, these findings suggest a novel mechanism of p53 malfunction in breast cancer cells, which is not related to the loss of p53, but to increased levels of the p53 isoforms. Of note, findings from protein overexpression cell models must be viewed with caution, since these cells do not represent the heterogeneity observed endogenously. However, our findings are in accordance with previous studies where the imbalanced ratio between p53 and its isoforms may predict worse prognosis in different cancers [6, 11, 25, 63, 64].

Given the striking differences between LeGO and Δ40p53 in vivo, we expected to observe opposing results with the stable knockdown of Δ40p53 as previously seen in vitro [20]. Instead, a slight increase (but not statistically significant) in DOX sensitivity was observed in -shΔ40p53 tumours compared to -shNT tumours (Fig. 5B). We have previously shown that stable knockdown of Δ40p53 leads to a differential transcriptional regulation at the basal level, upregulating genes such as *UBE2QL1* (a negative regulator of mTOR pathway) and increased proliferation [18]; however, following DOX, these cells showed increased apoptosis and G_1_ cell cycle arrest [20]. These results suggest that the depletion of Δ40p53 in unstressed cells may impair cells’ growth arrest [18] and affect mammary gland differentiation and development in normal breast cells (Fig. 4). Nevertheless, its silencing following DNA damage could enhance therapy efficacy [20]. Hence, the transient silencing of Δ40p53 in breast cancers may be more suitable to improve therapy response and target CSCs.

Enhanced therapeutic efficacy of DOX was observed when targeting Δ40p53 with concomitant chemotherapy (Fig. 7). Moreover, the decrease in DOX sensitivity in Δ40p53 cells can be bypassed with OTSSP167 treatment (Fig. 7), suggesting a promising combined therapy approach. Drugs such as OTSSP167 that target common pathways operating in cells with mutant or malfunctioning p53 and CSCs may have improved therapeutic effectiveness than those that solely target the p53 pathway [27]. For instance, MRX34, which is a mimic of miR-34, may restore some lost functions of mutant p53 [65, 66]. Hence, mimics of other miRNAs (e.g., miRNAs found downregulated in Δ40p53 cells) could possibly suppress the stemness phenotype in these cells. Along the same line, a compound named RETRA can disrupt mutant p53-p73 complexes restoring p73-dependent transcription and apoptosis [67]. A similar approach could be used to disrupt Δ40p53/p53 tetramers enhancing p53 function when Δ40p53 is highly expressed.

In light of these findings, we can speculate that high Δ40p53 levels in breast tumours (with wild-type *TP53*) trigger similar cell fate outcomes as loss-of-function p53 mutations. Increased Δ40p53 expression promotes p53 isoform stabilisation, upregulation of genes associated with pluripotency, and tumour growth contributing to chemoresistance in vivo. Our previous studies showed that Δ40p53 is highly expressed in a small percentage of breast cancer cells [25]. Nevertheless, these populations may drive treatment resistance [20] and tumour recurrence; thus, targeting Δ40p53 in breast cancer cells could be a novel approach to target CSCs and improve the canonical DNA damage response. Δ40p53 inhibition may result in opposing effects on normal and cancerous cells, but this requires further investigation.

## Materials and methods

### Study cohort

Total RNA extracted from 148 IDCs and hematoxylin and eosin (H&E) slides from 47 IDCs were provided by the Australian Breast Cancer Tissue Bank (Westmead, NSW, Australia) and have previously been described [6, 18, 68]. This study was conducted in accordance with the Helsinki Declaration with ethical approval from the Hunter New England Human Research Ethics Committee (approval number: 09/05/20/5.02) and the University of Newcastle Health and Safety Committee (approval number: R7/2021). All patients agreed to the use of their clinical information and tissue for research.

### Human Gene 1.0 Array

Human Gene 1.0 Array data (Affymetrix, Santa Clara, CA, USA) of 64 IDCs (GSE61725; patient information and clinical diagnoses are detailed in Supplementary Table 1) from our previous study [18] were reanalysed. The data were imported to Genomic Suite 7.0 (Partek) and a robust multi-array analysis (RMA) was performed as previously described [18]. Differential gene expression was evaluated in samples that had been grouped by their Δ40p53α (which is referred to as Δ40p53 for simplicity) expression level as high or low with median expression as the cut-off [6, 18] (*p* < 0.05; log_2_(fold change) >|1|). Correction for multiple testing was performed using the Benjamini-Hochberg procedure. Differentially expressed genes (DEGs) are detailed in Supplementary Tables 2 and 3.

Gene ontology (GO) analysis and heatmapping were performed in R (v.4.2.1) and associated R packages as detailed below [69]. GO (biological process) overrepresentation analysis was performed on DEGs (up or down) using enrichGO from clusterProfiler (v.4.4.4) [70] and visualised using enrichplot (v.1.16.1) [71]. Detected genes (from Array list) were used as the background list with false discovery rate (FDR) correction (adjusted *p* < 0.05). The most statistically significant pathways were summarised using the default settings calculated by ‘pairwise_termsim()’ on the GO results and displayed in a Tree plot with default hierarchical clustering by ‘treeplot()’. Heatmapping was performed using pheatmap (v.1.0.12) [72] on normalised counts from Partek filtered for DEGs with row (gene) normalisation by z-score followed by Euclidean hierarchical clustering of both columns (samples) and rows (genes). GO chord plot was plotted by https://www.bioinformatics.com.cn/en, an open-source data visualisation platform.

### Cell lines

The normal human epithelial breast cell line MCF-10A and the oestrogen receptor-positive human breast cancer cell lines MCF-7 and ZR75-1, expressing wild-type p53 (WTp53), were generously provided by A/Professor Nikki Verrills and Dr Rick Thorne, respectively. The cell lines were authenticated by the Australian Genome Research Facility as previously described [18]. MCF-7 cells stably overexpressing Δ40p53 via the lentiviral LeGO vector and MCF-7 and MCF-10A knockdown sublines, -shNT (non-targeting control), -shΔ40p53, and -shp53α, were established by transduction of cells with lentiviral vectors [18]. Each of the MCF-7 cell sublines and MCF-7 and ZR75-1 parental cells were maintained in DMEM (Dulbecco modified Eagle’s medium), supplemented with 10% foetal bovine serum (FBS), insulin (10 μg/mL), and L-glutamine (2 mM) (Life Technologies, Mulgrave, VIC, Australia). MCF-10A cell sublines were maintained in DMEM/F12 media supplemented with 10% horse serum, insulin (10 μg/mL), L-glutamine (2 mM), epidermal growth factor (20 ng/mL), hydrocortisone (0.5 μg/mL), cholera toxin (1 ng/mL) (Life Technologies). The medium used for the MCF-7 and MCF-10A cell sublines was further supplemented with puromycin (1 μg/mL) (Sigma-Aldrich, Castle Hill, NSW, Australia) for the maintenance of positive clones. Cells were maintained in humidified 5% CO_2_ at 37 ˚C and were routinely tested for mycoplasma according to the manufacturer’s recommendations (MycoAlert PLUS, Lonza, Basel, Switzerland).

### Immunofluorescence

Immunofluorescence was performed as previously described [20]. Cells were incubated for 1 h with primary antibodies: mouse-anti-human-Nanog (20 μg/ml; Life Technologies #MA1-017), mouse-anti-human-Sox2 (5 μg/ml; Life Technologies #MA1-014), mouse-anti-human-Oct4 (2 μg/ml; Life Technologies #MA1-104), rabbit-anti-human-Zeb1 (1 μg/ml; Bethyl Laboratories, Montgomery, TX, USA #A301-922A), rabbit-anti-human-p53 7F5 (1:800; Cell Signaling Technology, Danvers, MA, USA #2527), rabbit-anti-human-Δ40p53 KJC40 (detects all Δ40p53 isoforms, mainly Δ40p53α [18, 25]; 5 μg/ml; developed by J.C. Bourdon, The University of Dundee, Scotland), and/or rabbit-anti-human-GM130 (1:3200; Cell Signaling Technology #12480). Then, the cells were incubated for 1 h with secondary antibodies: goat-anti-mouse-Alexa Fluor 594 (1:30; Life Technologies #R37121), goat-anti-rabbit Alexa Fluor 594 (4 μg/ml; Life Technologies #A11037), and/or goat-anti-rabbit-Alexa Fluor 488 (4 μg/ml; Life Technologies #A11034). Each well was then stained with DAPI (300 nM in PBS) to detect cell nuclei. For cell spheroid immunofluorescence, following fixation, spheroids were sectioned with a cryostat and the slides were processed as described above. For breast cancer specimens immunofluorescence, FFPE IDC slides from our previous study looking at p53 isoform expression [25] were processed as previously described [73] with minor modifications (rinsing solution: 0.25% Triton-X-100 in phosphate-buffered saline (PBS) and blocking solution: 3% FBS in PBS). Three slides (S#1–3: ER+/PR+/Her2- IDCs) were incubated for 1 h with mouse-anti-human-CD38 (1:100; Leica Microsystems Pty Ltd, Mt Waverley, VIC, Australia #CD38-290-L-CE) and rabbit-anti-human-Δ40p53 KJC40 (8 μg/ml) antibodies and five slides of (S#4–7: ER+/PR+/Her2-and S#8: ER-/PR-/Her2+ IDCs) were incubated for 1 h with mouse-anti-human-Sox2 (5 μg/ml; Life Technologies #MA1-017) and rabbit-anti-human-Δ40p53 KJC40 (8 μg/ml) antibodies. Slides were then washed three times with the rinsing solution and incubated for 1 h with goat-anti-mouse-Alexa-Fluor 594 (1:30) and goat-anti-rabbit-Alexa 488 (4 μg/ml) secondary antibodies. After the final rinsing steps, mounting medium with DAPI was added to the slides. Images were obtained using a Cytation3 cell imager (BioTek, Winooski, VT, USA) using 10x and 40x objectives. For co-localisation analysis in MCF-7 and ZR75-1 cells and IDCs slides, four images were collected per well (approximately 30 cells were evaluated per triplicate) and ten microscope fields were collected per slide, respectively. Images were collected maintaining exposure and contrast settings. Images were analysed using Gen5 software for fluorescence intensity and ImageJ (Coloc 2) for co-localisation, which performs a pixel intensity correlation of regions of interest. Spearman’s rank correlation was calculated between Δ40p53 or p53 and Nanog, Sox2, or Oct4. The identification of the images was blinded to the investigator.

### RNA extraction

Total RNA was extracted from cell lines using TRIzol RNA purification reagent (Life Technologies) following manufacturer’s recommendations. The RNA yield was determined by the Qubit RNA BR (broad range) Assay Kit (Life Technologies) on a Qubit 2.0 Fluorometer (Life Technologies), following manufacturer’s recommendations.

### Reverse transcription quantitative polymerase chain reactions (RT-qPCR)

Total RNA of 148 IDCs (500 ng) and cell line samples (300 ng) was reverse transcribed into complementary DNA (cDNA) using the High-Capacity Reverse Transcription kit with RNase inhibitor (Life Technologies), as per the manufacturer’s instructions. No template RNA and no reverse transcriptase controls were included. TaqMan Advanced Master Mix (Life Technologies) and TaqMan Gene Expression assays for *KI67* (Mm01278617_m1), *NANOG* (Hs02387400_g1), *OCT4* (At02611156_m1), *SOX2* (Hs04234836_s1), *ZEB1* (Hs00232783_m1), *VIM* (Hs00185584_m1), *CDH1* (Hs01023894_m1), *SNAI1* (Hs00195591_m1), *SNAI2* (Hs00161904_m1), and *Δ40p53* (as previously described [6]) were used. *β-actin* (Hs01060665_g1) and *GAPDH* (Hs02786624_g1) were used as endogenous controls. Relative expression was calculated using the 2^−ΔΔCt^ method [74]. Gene expression analysis of miRNAs was performed using TaqMan Advanced miRNA cDNA Synthesis Kit according to manufacturer’s recommendations (Life Technologies). TaqMan Advanced Master Mix (Life Technologies) and TaqMan Gene Expression assays for *hsa-miR-145-5p* (477916_mir), *hsa-miR-200b-3p* (477963_mir), and *hsa-miR-200c-3p* (478351_mir) were used. *Hsa-miR-16-5p* (477860_mir) was used as an endogenous control [75].

### Gene expression analysis of single cells

MCF-7-LeGO and -Δ40p53 single cells were captured using Fluidigm integrated fluidic circuits with preamplification using TaqMan Assays on the Fluidigm C1 system (Fluidigm, South San Francisco, CA, USA) as per the manufacturer’s instructions. Single-cell qPCR of stem cell markers: *SOX2* (Hs04234836_s1)*, OCT4* (At02611156_m1)*, NANOG* (Hs02387400_g1)*, ELF5* (Hs01063022_m1)*, ITGA6* (Hs01041011_m1)*, ITGB1* (Hs00559595_m1), and *FOXM1* (Hs01073586_m1), and the endogenous control *GAPDH* (Hs02786624_g1) was performed using Biomark HD System with 96.96 Dynamic Array integrated fluidic circuits (Fluidigm, USA). Data was analysed using Singular Analysis Toolset build under R and data are visualised using violin plot.

### Immunoblotting

Proteins were separated by sulphate dodecyl sulphate-polyacrylamide gel electrophoresis (SDS-PAGE) as previously described [18]. The membrane was blocked with Casein Blocking Buffer (Millennium Science, Mulgrave VIC, Australia) at room temperature for 1 h. The following primary antibodies were diluted in blocking buffer: pan-p53 rabbit-anti-human-CM-1 (1 μg/ml; The University of Dundee, Scotland), rabbit-anti-human-Nanog (1 μg/ml; Cell Signaling Technology #D73G4), rabbit-anti-human-Sox2 (1 μg/ml; Cell Signaling Technology #D609), rabbit-anti-human-β-catenin (1 μg/ml; Abcam, Melbourne, VIC, Australia #ab32572), rabbit-anti-human-Δ40p53 KJC40 (2.5 μg/ml), and mouse-anti-human-GAPDH (1 μg/ml; Calbiochem, San Diego, CA, USA #CB1001), and added to the membrane overnight (4 °C, rocking). Diluted secondary antibodies (1–5 μg/ml; LI-COR Biosciences, Lincoln, NE, USA) in blocking buffer were added and allowed to bind on a rocker for at least 1 h at room temperature. Bands were visualised and quantitated using an Odyssey CLx fluorescent imager (LI-COR Biosciences) relative to the loading control (GAPDH). Uncropped membranes are shown in the original western blots Supplemental file.

### Flow cytometry and fluorescence-activated cell sorting (FACS) analysis

1 × 10^6^ cells of each MCF-7-LeGO and -Δ40p53 sublines were resuspended in 100 μL of ice-cold 2% FBS in PBS containing allophycocyanin (APC)-conjugated mouse-anti-human CD44 (20 μL; clone G44-26; BD Biosciences, Becton Dickinson Pty Ltd, Macquarie Park, NSW, Australia #550392), BD Horizon Brilliant Violet 421 (BV421)-conjugated mouse anti-human CD24 monoclonal antibody (20 μL; clone ML5; BD Biosciences #562789), and 7-amino-actinomycin D (7-AAD) for 20 min at 4 °C in the dark. The cells were then rinsed twice with 2% FBS in PBS. CD44 and CD24 levels were determined using a BD FACS Aria III flow cytometer (BD Biosciences). Unstained cells were used as negative controls.

### Mammospheres formation assay

For mammosphere formation, 1 × 10^3^ cells/well were plated in 24-well ultra-low attachment plates (Corning, NY, USA) with MammoCult medium enriched with MammoCult proliferation supplement (10% v/v), hydrocortisone (0.48 µg/ml), and heparin (4 µg/ml) (StemCell Technologies, Vancouver, BC, Canada) and cultured for 7 days. After 7 days of culture the size and number of formed mammospheres were quantified using a Cytation3 cell imager (BioTek).

### Colony formation and cell spheroid assays

For the colony formation assays, 1 × 10^3^ cells/well were seeded onto 6-well plates. Cells were grown for 14 days, at which time visible colonies were apparent. Cells were fixed with ice-cold methanol and stained with 0.5% crystal violet solution. Colony number and size were calculated using the cellSens Standard software (Olympus, Notting Hill, VIC, Australia). For the cell spheroid assay, cells were seeded in 96-well ultra-low attachment plates (Corning) at 4 × 10^3^ cells/well and the plates were centrifuged for 5 min at 200 × *g* to allow the formation of cell spheroids. The spheroids were imaged after 7 days of culture using a Cytation3 cell imager (BioTek).

### Acini formation assay

Three-dimensional acinar assay in extracellular matrix (ECM; Sigma-Aldrich) was performed as previously described [76]. Briefly, 45 µl of ECM was carefully dispensed into 8-well chamber slides and allowed to solidify for at least 30 min in a standard cell culture incubator. MCF-10A cell sublines were trypsinised, washed, and resuspended in complete medium. 5 × 10^3^ cells/well were seeded onto 8-well chamber slides (200 µl/well), and the chamber slides were returned to the cell culture incubator for 30 min for the cells to set onto the precoated ECM. 4% ECM in 200 µl of complete media (for a final concentration of 2% ECM/well) was carefully dispensed into each well. The media containing 2% ECM was replenished every 2 days. Every 7 days of culture, the size and number of formed acinar structures were quantified using a Cytation3 cell imager (BioTek). A threshold of 20 µm was set to filter out cell debris as the size of a MCF-10A cell is ~20 µm. All acini were processed for phalloidin staining after 21 days in culture. Briefly, acini were fixed with 3.7% formaldehyde (Sigma-Aldrich) in PBS for 10 min. The acini were subsequently incubated with TRITC-conjugated phalloidin (10 μg/ml; Sigma-Aldrich) for 20 min. The nuclei were stained with DAPI and mounted using ProLong Gold Antifade mount media (Life Technologies) on glass slides. Confocal microscopy was performed using a Leica DMRE upright fluorescent microscope (Leica, Wetzlar, Hesse, Germany) fitted with a blue argon (488 nm—FITC excitation) and a green helium neon (568 nm—TXR excitation) laser and a 20x objective.

### Luciferase lentiviral particles transfection

For in vivo imaging purposes, MCF-7 sublines were transfected with luciferase lentiviral particles according to the manufacturer’s recommendation (GenTarget Inc., San Diego, CA, USA). Briefly, cells were seeded at 2 × 10^5^ cells/well in 6-well plates until they reached 50% of confluence. Next, 500 µL of complete medium and 50 µL of lentiviral particles were added to each well. Media were replenished every 3–4 days with media containing the selection antibiotic, G-418 (400 µg/mL; Sigma-Aldrich), until resistant colonies were identified.

### Engraftment of NOD scid gamma mice

Six-week-old female NOD scid gamma (NSG) mice were obtained from the Animal Resources Centre (ARC, Murdoch, WA, Australia), and were kept at the Hunter Medical Research Institute Bioresources Facility at 22 ± 2 °C, with water and food *ad libitum*, and under a 12:12 h light and dark photoperiod. All experimental procedures were reviewed, approved, and carried out according to the Animal Care and Ethics Committee of the University of Newcastle (approval number: A-2020-016). G*Power 3.1 [77] was used to perform calculations on sample size, effect size, and statistical power. The minimal significance (α) and statistical power (1-β) were set at 0.05 and 0.80, respectively. Calculations were carried out for two groups by using Student’s t-distribution. The NSG mice were orthotopically injected with luciferase-labelled MCF-7 sublines (-shNT, -shΔ40p53, LeGO, or Δ40p53) at 2 × 10^6^ cells suspended in 50:50 matrigel matrix phenol red-free high concentration (Corning)-PBS into the mammary fat pad under isoflurane anaesthesia. Simultaneously, the mice were implanted subcutaneously at the back of the neck with 17β-oestradiol pellets (60-day release, 0.36 mg/pellet; Innovative Research of America, Sarasota, FL, USA). The animals were monitored daily and body weight was recorded twice a week with a digital balance. The tumour take rate was found to be 50%. Tumour burden was assessed twice a week using digital calliper measurements (tumour volume = (length × width × depth)/2). For in vivo imaging, subcutaneous injections of D-luciferin (Sapphire Bioscience, Redfern, NSW, Australia) at a dose of 150 mg/kg were administered to animals under isoflurane anaesthesia. The luminescence signal was recorded using an in vivo imaging system (Xenogen IVIS 100 bioluminescent in-vivo imaging system, PerkinElmer, Waltham, MA, USA). Once tumours were established (50–100 mm^3^), the treatment regimen was started as detailed below.

### In vivo treatment

Treatment 1: Three different doses of DOX were administered via repeated weekly intravenous injections to mice bearing the luciferase-labelled MCF-7-shNT subline to select the best tolerated dose that resulted in a reduction in tumour size. Randomly allocated mice were treated by tail vein intravenous injection with either vehicle (saline) (*n* = 3), or three different doses of DOX (1 mg/kg, 2 mg/kg, or 3 mg/kg; *n* = 3 in each treatment group) under isoflurane anaesthesia once a week for 3 weeks. The animals were followed up for 7 days after the last treatment, or until they reached ethical end-point (moribund animal and/or loss of >10% of initial body weight). All animals were euthanised by carbon dioxide asphyxiation (Supplementary Fig. 9A).

Treatment 2: Mice engrafted with luciferase-labelled sublines were randomly divided into eight groups (*n* = 5–9 mice/group): -shNT (vehicle or DOX-treated), -shΔ40p53 (vehicle or DOX-treated), LeGO (vehicle or DOX-treated), and Δ40p53 (vehicle or DOX-treated). DOX (2 mg/kg) (Supplementary Fig. 9A) or vehicle (saline) were administered once a week for 3 weeks, by intravenous tail vein injection under isofluorane anaesthesia. The identification of the mice (i.e., subline engrafted) was blinded to the investigator during treatment. The animals were monitored daily for clinical and behavioural changes and biweekly for body weight changes (Supplementary Fig. 9B). The animals were followed up for 7 days after the last treatment, or until they reached ethical end-point (moribund animal and/or loss of >10% of initial body weight). All animals were euthanised by carbon dioxide asphyxiation. After euthanasia, the tumours and spleens were harvested and preserved in buffered formalin solution (10%, pH 7.4; Sigma-Aldrich).

### Histological analysis

Specimen processing and H&E staining were performed by the Hunter Medical Research Institute Core Histology Facility (Newcastle, NSW, Australia) according to established protocols. Immunohistochemistry (IHC) was performed by the NSW Regional Biospecimen & Research Services (Newcastle, NSW, Australia) using a Ventana Discovery Automated Immunostainer (Roche Medical Systems, Tuscon, AZ, USA) as previously described [25]. Tissue sections (4 µm/section) were deparaffinised and incubated in a Ventana solution for antigen retrieval at pH 9. After antigen retrieval, slides were incubated for 12 min with a peroxidase inhibitor (Roche Medical Systems) followed by incubation with mouse-anti-human-Sox2 (2.5 μg/ml; Life Technologies #MA1-014) or rabbit-anti-human-Ki67 (0.07 μg/ml; Life Technologies #MA5-14520) primary antibodies for 32 min at 37 °C (for slides of positive controls used during antibody optimisation see Supplementary Fig. 10). The pre-diluted anti-mouse hapten (HQ) or anti-rabbit HQ secondary antibodies (Roche Medical Systems) were added and slides were incubated with anti-HQ-horseradish peroxidase (HRP) (Roche Medical Systems), and visualised using diaminobenzidine (DAB) chromogen detection kit (Roche Medical Systems). All slides were manually counterstained with Mayers hematoxylin. Slides were scanned at 40x magnification using an Aperio AT2 scanner (Leica, Wetzlar, Germany), and analysed with HALO Software (Halo imaging analysis software, Indica Labs, Corrales, NM, USA) using the CytoNuclear v2.0.8 analysis mode. H-scores and the percentage of positive cells [7] were quantified. For microvessel area quantification, an analysis mask was created to recognise blood vessels based on the H&E staining (morphology/colour). Tissue artefacts were excluded from the analysis.

H&E slides of the IDC cohort (*n* = 47) were used to assess TILs. TILs were visualised using HALO Software and manually quantified according to [78].

### siRNA transfection

Two siRNAs targeting intron 2 of *TP53* were used to knockdown Δ40p53: Δ40p53 #1 (5′-AGACCTGTGGGAAGCGAAA-3′) and Δ40p53 #2 (5′-GCGAAAATTCCATGGGACT-3′) in MCF-7 cells. A non-targeting siRNA was used as a control (D-001810-01-20) (Horizon Discovery, Dharmacon, Millenium Science). For confluence assays, 1 × 10^4^ cells/well were seeded into 96-well plates. For downstream protein expression analysis, 3 × 10^5^ cells/well were seeded in 6-well plates. siRNAs were diluted with 1 x siRNA buffer and serum-reduced media (Opti-MEM) and mixed with DharmaFECT transfection reagent-1 (Millennium Science) diluted in Opti-MEM (Life Technologies). The mixture was diluted in pre-warmed media to achieve a final concentration of 25 nM siRNA. Cells were then treated with DOX (1 µM). For protein analysis, cells were harvested following 24 h of treatment.

### Cell confluence and death assay

For real-time cell death assessment and confluence analysis, propidium iodide (PI) at the final concentration of 2.5 µg/mL (Sigma-Aldrich) was added to the wells as previously described [79]. Cells were then placed into an incubator connected to an IncuCyte imaging system (Sartorius, Göttingen, Germany). Images were analysed using the IncuCyte Zoom software. Cell confluence and PI-positive cells normalised to confluence were calculated using integrated software algorithms.

### OTSSP167 and DOX treatment

Cells were treated with DOX (1 µM) and/or the Melk inhibitor, OTSSP167 (40 nM) (Selleckchem, Houston, TX, USA). The effects of the treatments were assessed by mammosphere and cell spheroid formation assays as described above. For the mammosphere assay, the size and number of formed mammospheres were quantified after 7 days of treatment. For the cell spheroid assay, spheroids were imaged every other day and on the seventh day of treatment, half of the media was replenished and additional treatment was added to the wells. After 14 days of treatment, spheroid size was recorded and spheroid viability was evaluated using CellTiter-Glo 3D assay as per manufacturer’s recommendations (Promega, Madison, WI, USA). Images of the created mammospheres, spheroids, and luminescence was recorded using a Cytation3 cell imager (BioTek) and analysed using Gen5 software.

### Statistical analysis

All continuous variables were tested for normal distribution. Unpaired student t-tests or Mann–Whitney tests were performed for two comparisons. For multiple comparisons, one-way ANOVA corrected for multiple comparisons using the Dunnett’s or Tukey’s tests or two-way ANOVA corrected for multiple comparisons using the Sidak’s or Tukey’s tests were performed. All results are the mean of three independent experiments, and error bars represent the standard deviation (SD) or the standard error of the mean (SEM). Spearman rank correlation analysis was used to compare the relative mRNA expression of Δ40p53 with Ki67 in tumour tissues. All statistical analyses were performed using GraphPad Prism v. 9.0 (GraphPad Software, La Jolla, CA, USA). An adjusted *p*-value of <0.05 was considered statistically significant.

## Supplementary information

Original Data File

Supplemental Material

Supplementary Table S1

Supplementary Table S2

Supplementary Table S3

Supplementary Table S4

Supplementary Table S5

## Data Availability

Data from HumanGene1.0 Arrays were deposited in the NCBI Gene Expression Omnibus database with the accession number GSE61725. Other data generated in this study are available within the supplementary data files or upon request from the corresponding author.
