# Supplementary figures and images for: p53 isoform expression promotes a stemness phenotype and inhibits doxorubicin sensitivity in breast cancer
Source: Cell Death Dis. 2023 Aug 8;14(8):509. doi: 10.1038/s41419-023-06031-4 (PMC10409720; doi:10.1038/s41419-023-06031-4)

Figure 3C

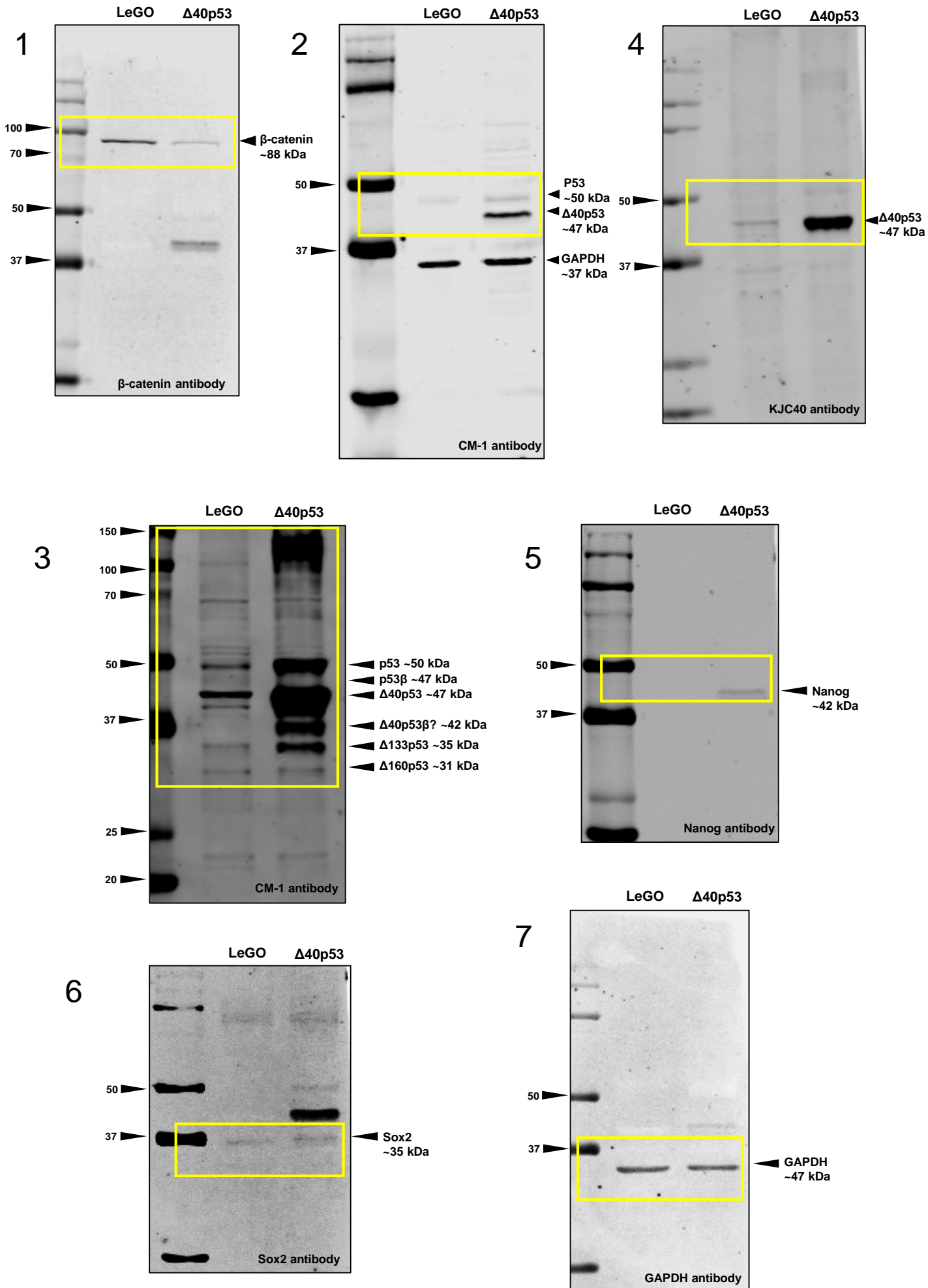

Figure 4B

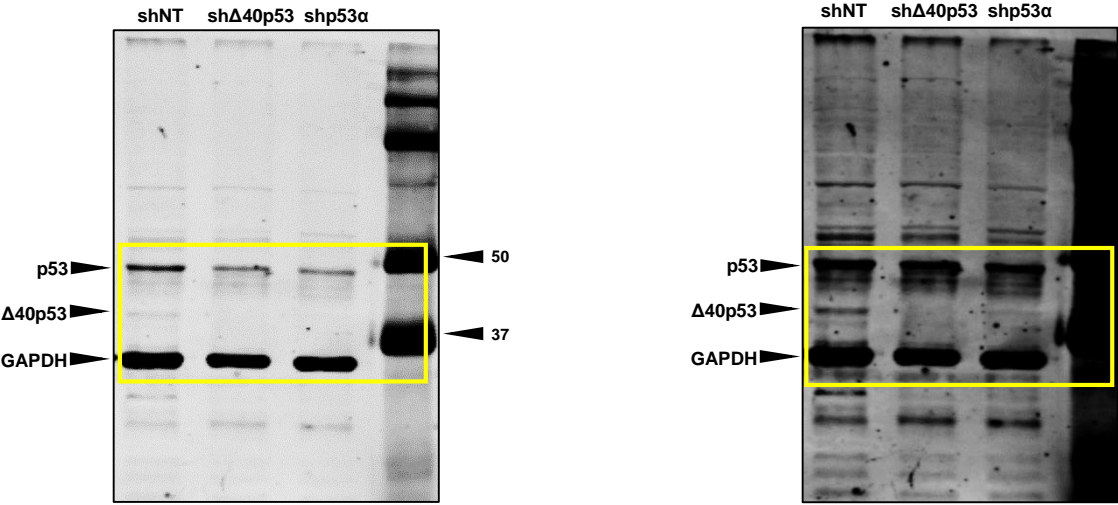

Figure 7A

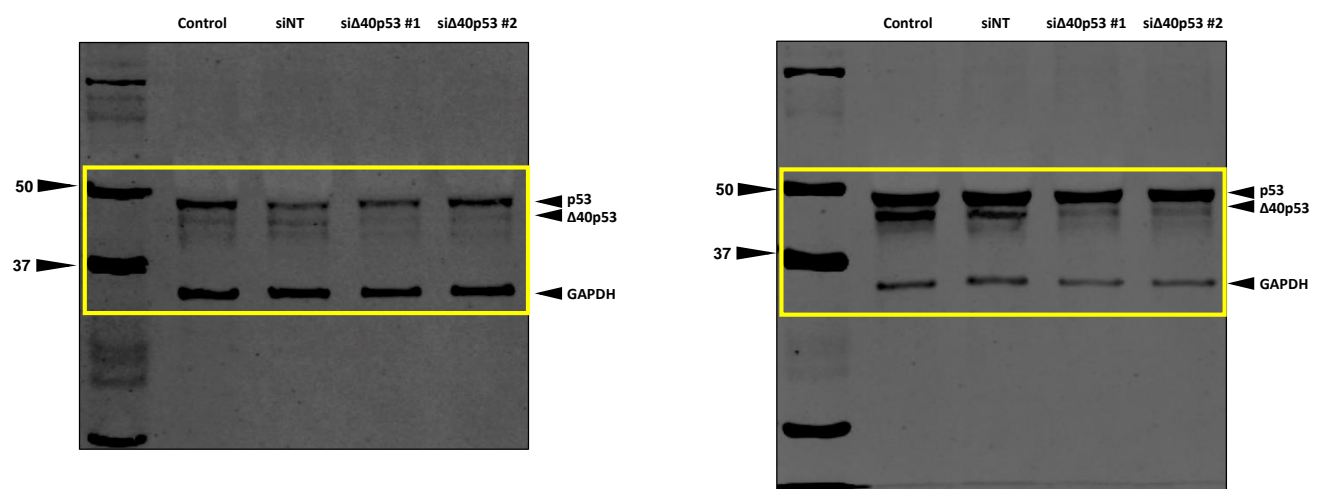

Supplement: Supplementary file 1 — Original Data File [file 41419_2023_6031_MOESM1_ESM.pdf]
