# Supplementary material for: p53 isoform expression promotes a stemness phenotype and inhibits doxorubicin sensitivity in breast cancer
Source: Cell Death Dis. 2023 Aug 8;14(8):509. doi: 10.1038/s41419-023-06031-4 (PMC10409720; doi:10.1038/s41419-023-06031-4)
Supplement: Supplementary file 2 — Supplemental Material [file 41419_2023_6031_MOESM2_ESM.pdf]

Supplementary

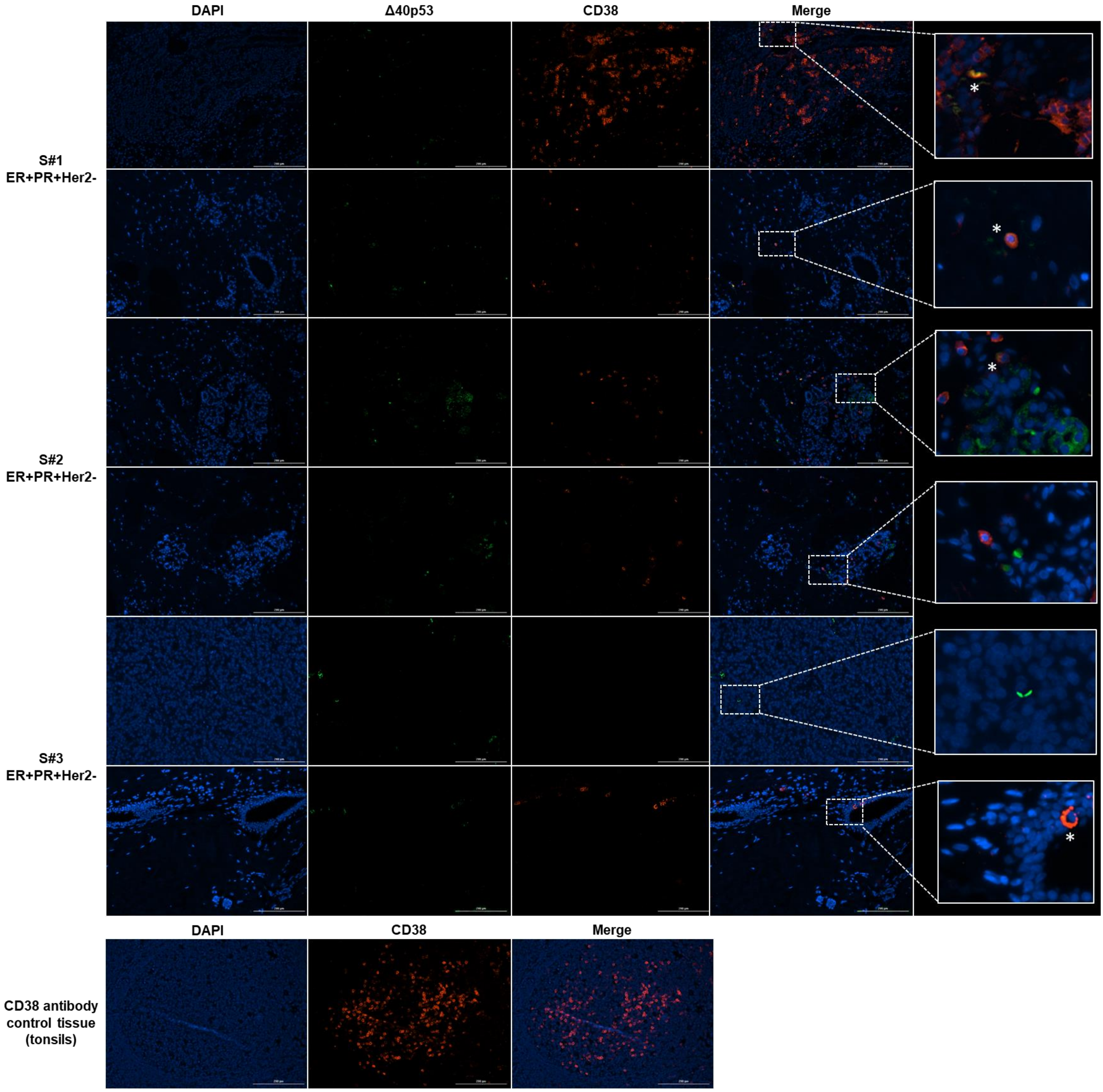

Figure S1: Δ40p53 is expressed in breast cancer cells and plasma cells in invasive ductal carcinoma (IDC) specimens. Immunofluorescence images of Δ40p53 and CD38 co-staining in three IDC specimens (S#1-3). Δ40p53 (KJC40) (8 µg/ml) and plasma cell marker CD38 (1:100) primary antibodies were used and cell nuclei were stained with DAPI. Far right pictures demonstrate positive staining for Δ40p53 and/or CD38. * shows cells where Δ40p53 and CD38 co-localised. Scale bar indicates 200 µm. Human tonsils were used as a positive control for the CD38 staining. S: specimen.

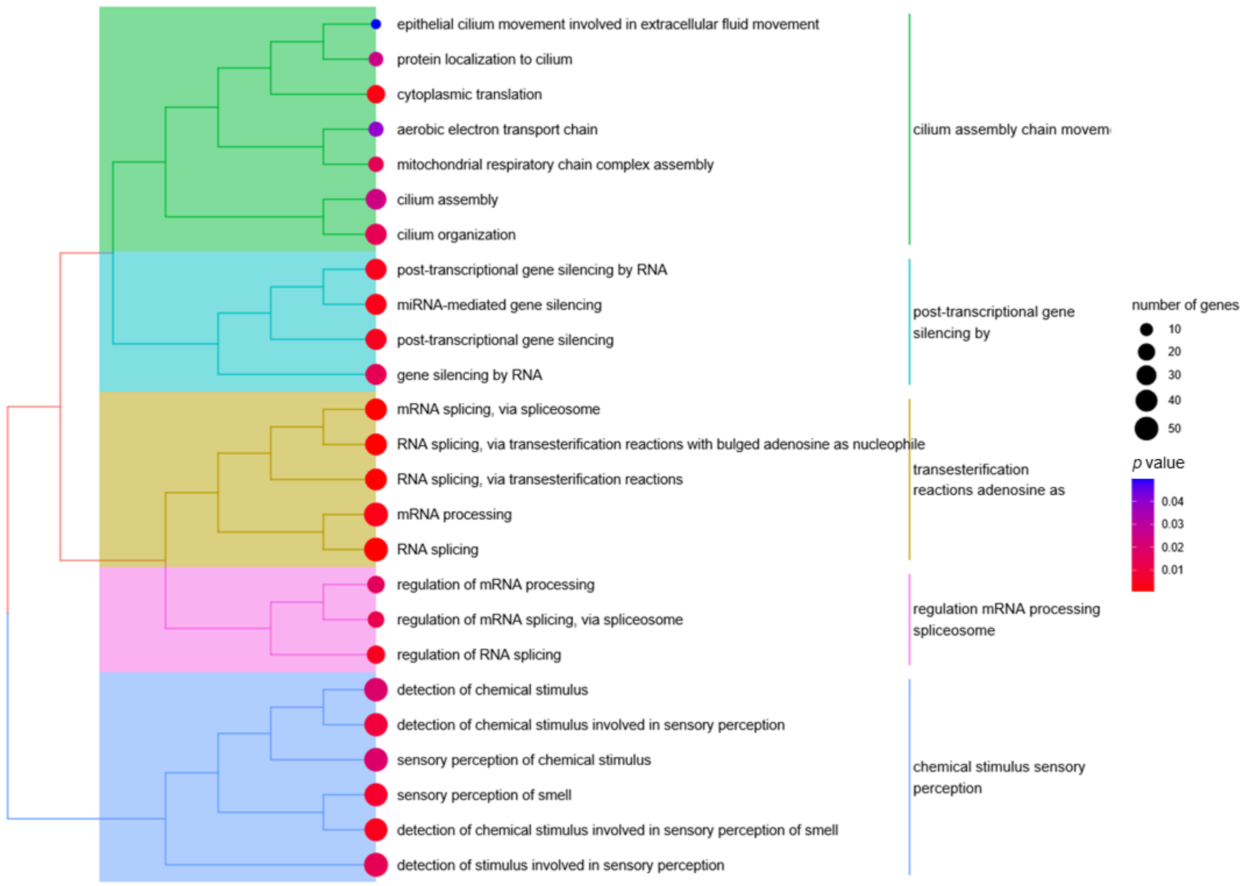

Figure S2: Tree plot of top 30 gene ontology (GO) terms enriched in upregulated genes in high Δ40p53 versus low invasive ductal carcinomas (IDCs).

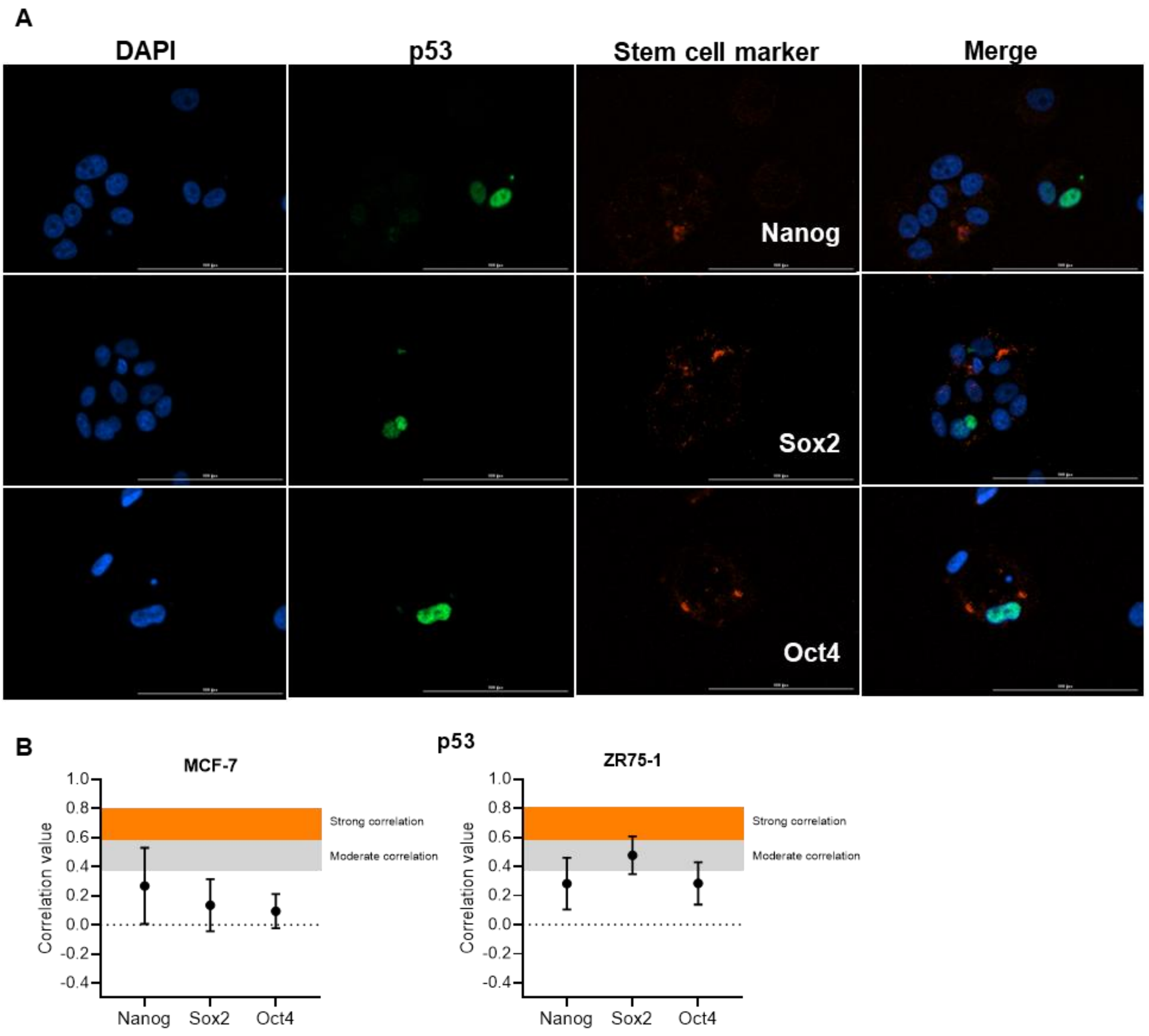

Figure S3: p53 does not co-localise with stem cell markers, Nanog, Sox2, and Oct4. (A) Immunofluorescence images of p53 and Nanog, Sox2, or Oct4 co-staining in the MCF-7 cell line. p53 (7F5) (1:800) and stem cell markers Nanog (20 µg/ml), Sox2 (5 µg/ml), and Oct4 (2 µg/ml) primary antibodies were used and cell nuclei were stained with DAPI. Scale bar indicates 100 µm. (B) Correlation values of the co-localisation of p53 and Nanog, Sox2, or Oct4 in MCF-7 and ZR75-1 cells. Analyses were carried out using ImageJ (Coloc 2) and Spearman's rank correlation was used for the co-localisation analyses.

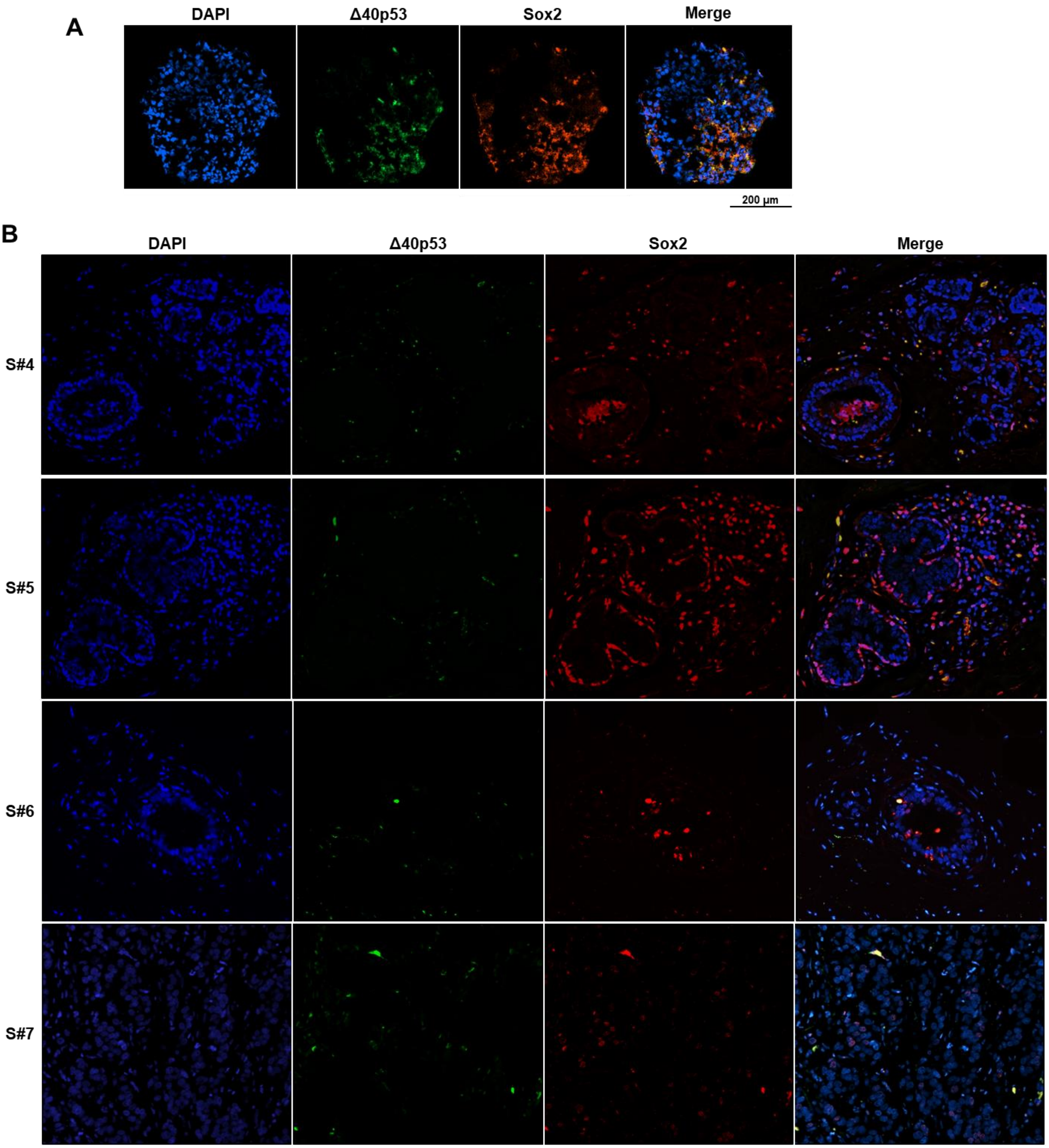

Figure S4: Single staining of Figure 2C and D: Δ40p53 co-localisation with Sox2. *(*A) Immunofluorescence images of Δ40p53 and Sox2 staining in a ZR75-1 cell spheroid and (B) in five breast cancer specimens (S#4-7). KJC40 (5 µg/ml (A) and 8 µg/ml (B)) and Sox2 (5 µg/ml) primary antibodies were used and cell nuclei were stained with DAPI. S: specimen.

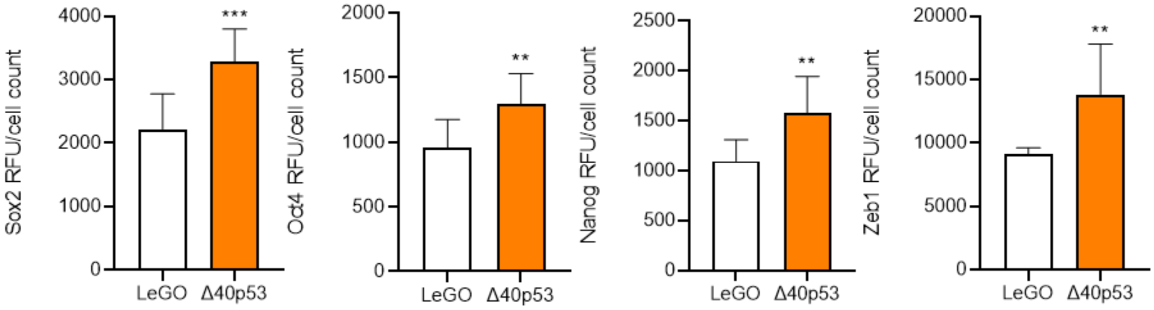

Figure S5: Sox2, Oct4, Nanog, and Zeb1 expression in relative fluorescence units (RFU) normalised to cell count in MCF-7-LeGO and MCF-7-Δ40p53 cells. Data shown represent three independent experiments of three technical replicates. Results are shown as the mean ± SD. Statistical analyses were carried out using an unpaired t-test. Results were considered significant at $p < 0.05$; **$p < 0.01$, ***$p < 0.001$.

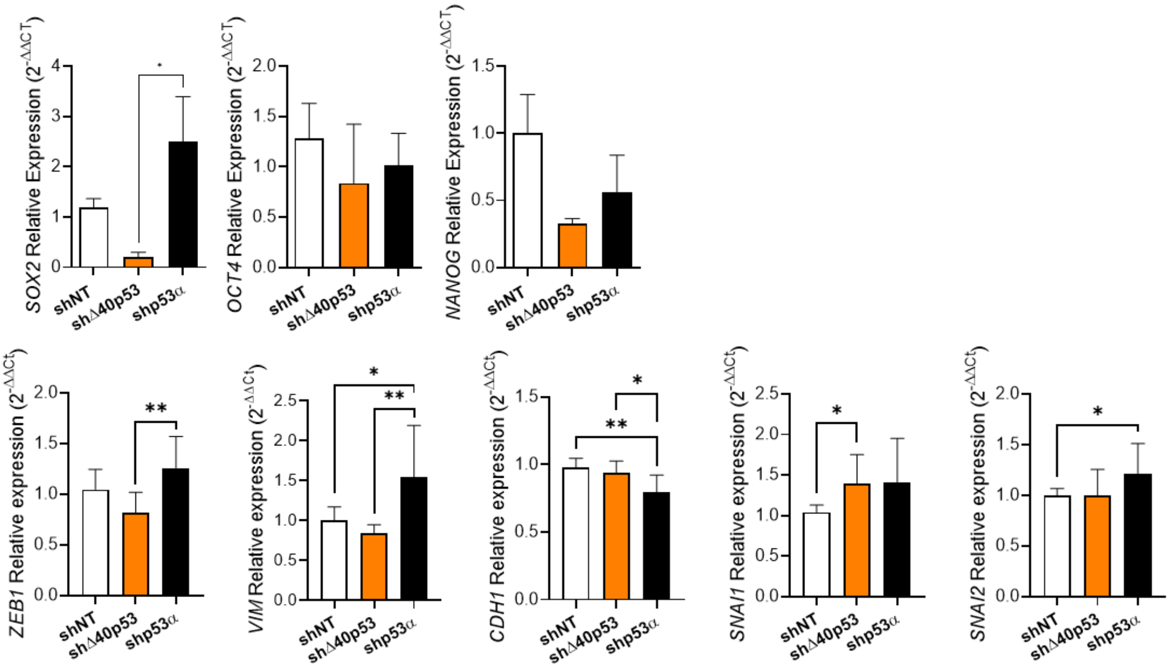

Figure S6: mRNA levels of *SOX2*, *OCT4*, *NANOG*, *ZEB1*, *VIM*, *CDH1*, *SNAI1*, and *SNAI2* in MCF-7-shNT, MCF-7-shΔ40p53, and MCF-7-shp53 sublines. Data shown represent three independent experiments of three technical replicates. Results are shown as the mean ± SD. Statistical analyses were carried out using one-way ANOVA followed by Tukey's post-test. Results were considered significant at $p < 0.05$; *$p < 0.05$, **$p < 0.01$.

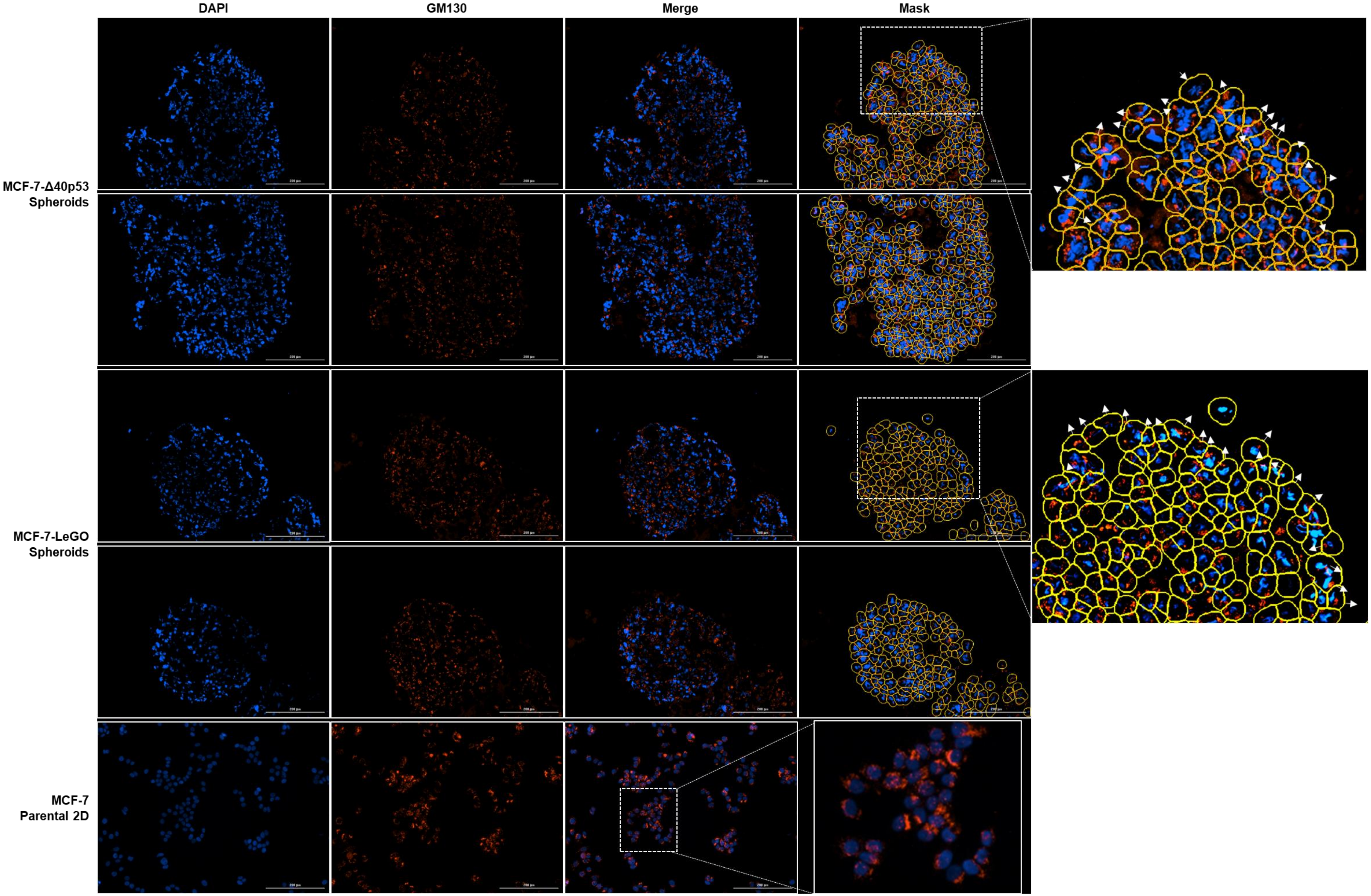
DAPI
GM130
Merge
Mask
MCF-7-Δ40p53
Spheroids
MCF-7-LeGO
Spheroids
MCF-7
Parental 2D

Figure S7: Golgi orientation of MCF-7-LeGO and MCF-7-Δ40p53 cell spheroids. Immunofluorescence images of GM130 staining in the cell spheroids. GM130 (1:3200) primary antibody was used and cell nuclei were stained with DAPI. Far right pictures: Cytation3 was used to draw cell masking. Arrows indicate the direction of the polarity determined by the position of the GM130 staining. GM130 staining in MCF-7 cells was performed for comparison reasons.

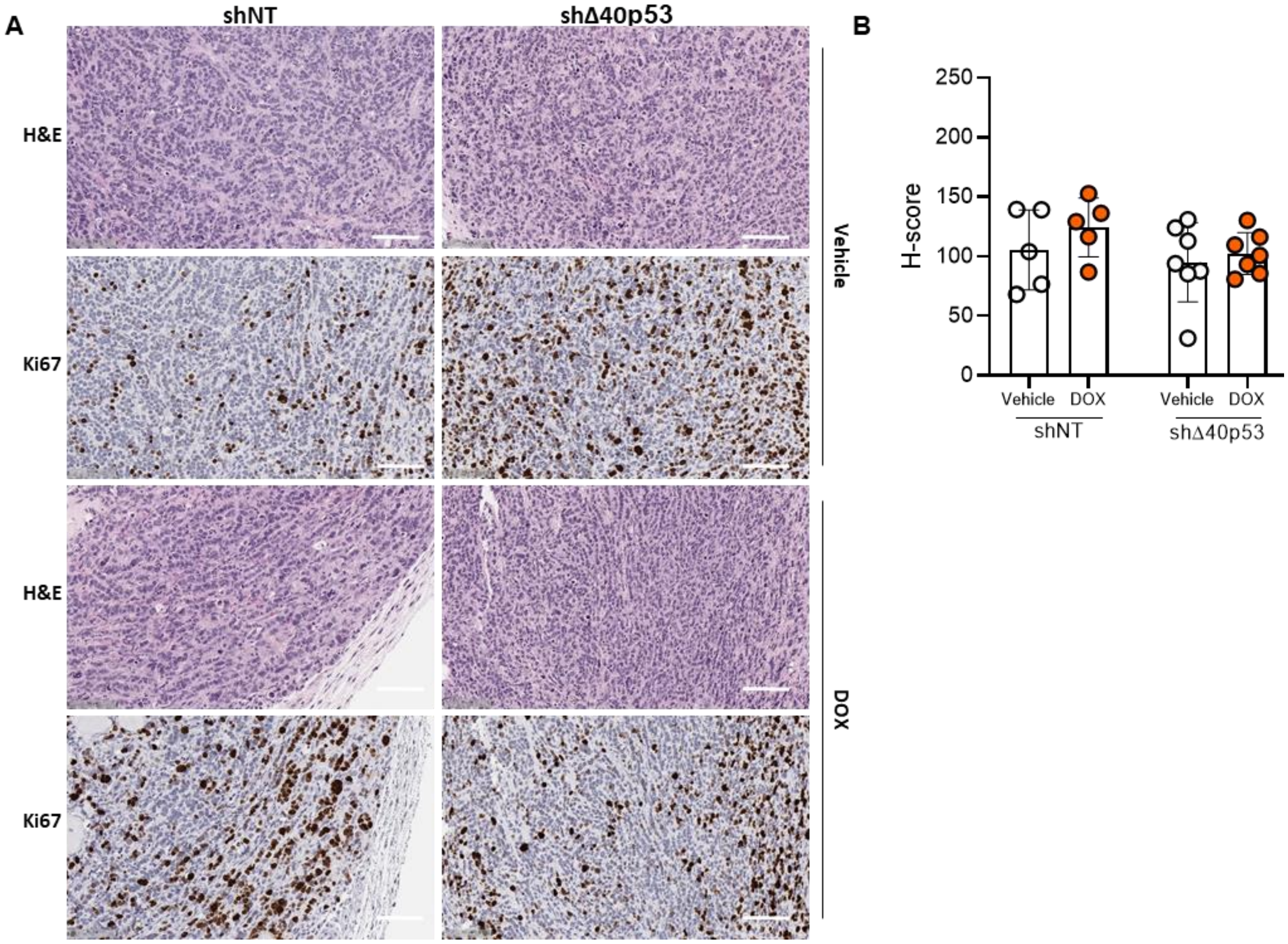

Figure S8: Ki67 levels in Δ40p53 knockdown tumour xenografts. (A) Representative images of H&E slides and slides stained for Ki67 of MCF-7-shNT or MCF-7-shΔ40p53 tumour-bearing mice treated with vehicle or DOX. Scale bars represent 100 µm. Ki67 (0.07 µg/ml) primary antibody was used. (B) H-score for Ki67 in MCF-7-shNT or MCF-7-shΔ40p53 xenograft tumours. Results are shown as the mean ±SD. Statistical analysis were carried out using two-way ANOVA followed by Sidak's post-test. Results were considered significant at $p < 0.05$.

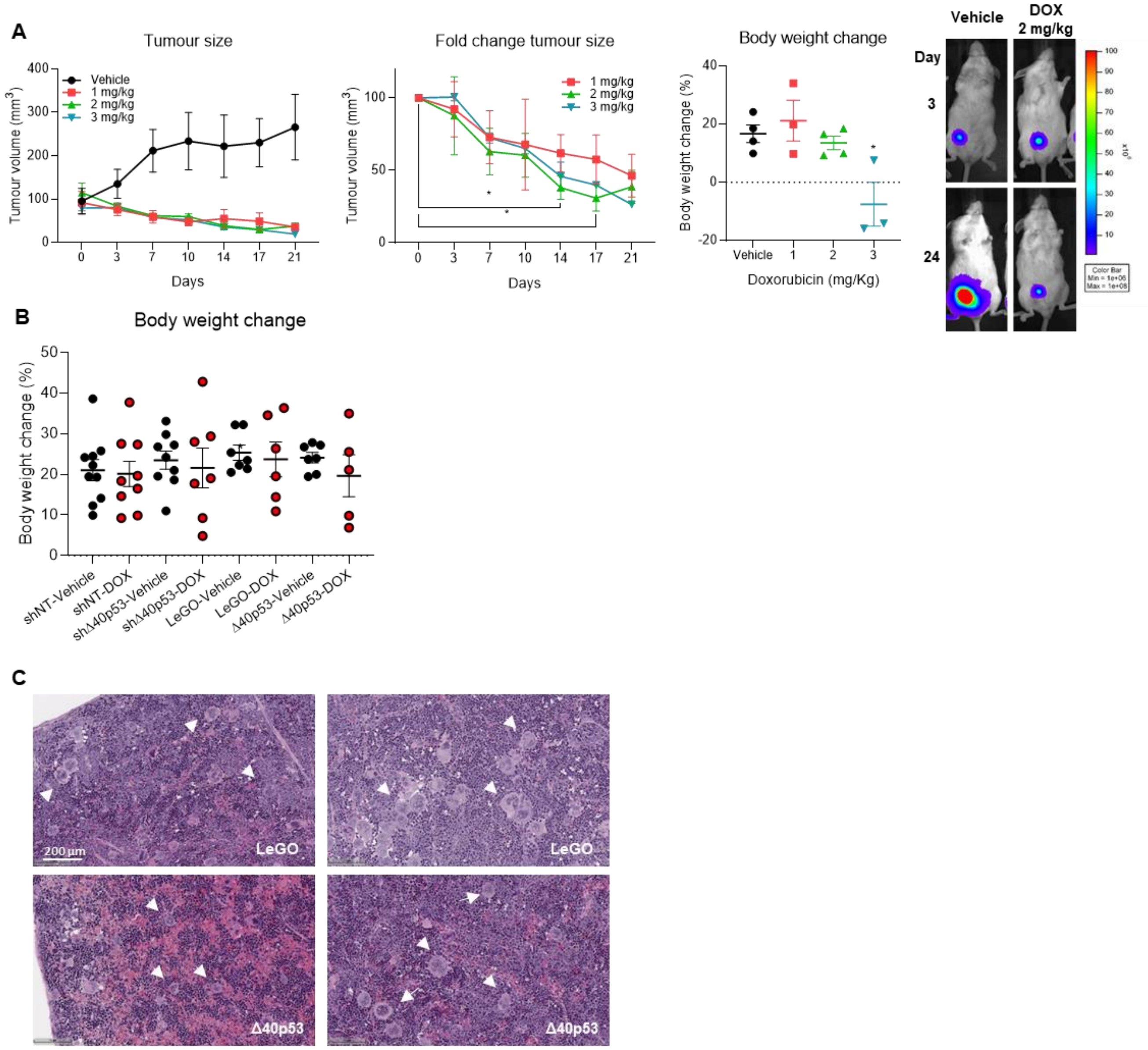

Figure S9: *In vivo* experiments. (A) Pilot *in vivo* study (see Methods – Treatment 1) to select the suboptimal dose of DOX. Tumour volume normalised to tumour size on day 0 (prior treatment) and body weight change of mice bearing the luciferase-labelled MCF-7-shNT subline treated with saline or three different doses of DOX (1 mg/kg, 2 mg/kg, or 3 mg/kg) (n = 3 in each treatment group). Representative images of luminescent imaging of mice treated with saline or DOX (2 mg/kg). DOX at 2 mg/kg was selected for the Treatment 2 (see Methods). (B) Body weight change of MCF-7-shNT, MCF-7-shΔ40p53, MCF-7-LeGO, and MCF-7-Δ40p53-derived xenografts treated with saline (vehicle) or DOX (2 mg/kg). (C) Representative H&E images of spleens that presented luciferin signal during mice imaging. Breast cancer cells were observed in the spleens (white arrows). These cells escaped the mammary fat pad during implantation of cells and were detected in the spleens due to splenic clearance. No differences were observed between MCF-7 sublines groups. No additional luminescence signals were detected during *in vivo* imaging. Scale bars represents 200 µm. Results are shown as the mean ± SD. Statistical analyses were carried out using one-way ANOVA followed by Dunnett's post-test (body weight change) or two-way ANOVA followed by Tukey's post-test (tumour volume). Results were considered significant at $p < 0.05$; *$p < 0.05$.

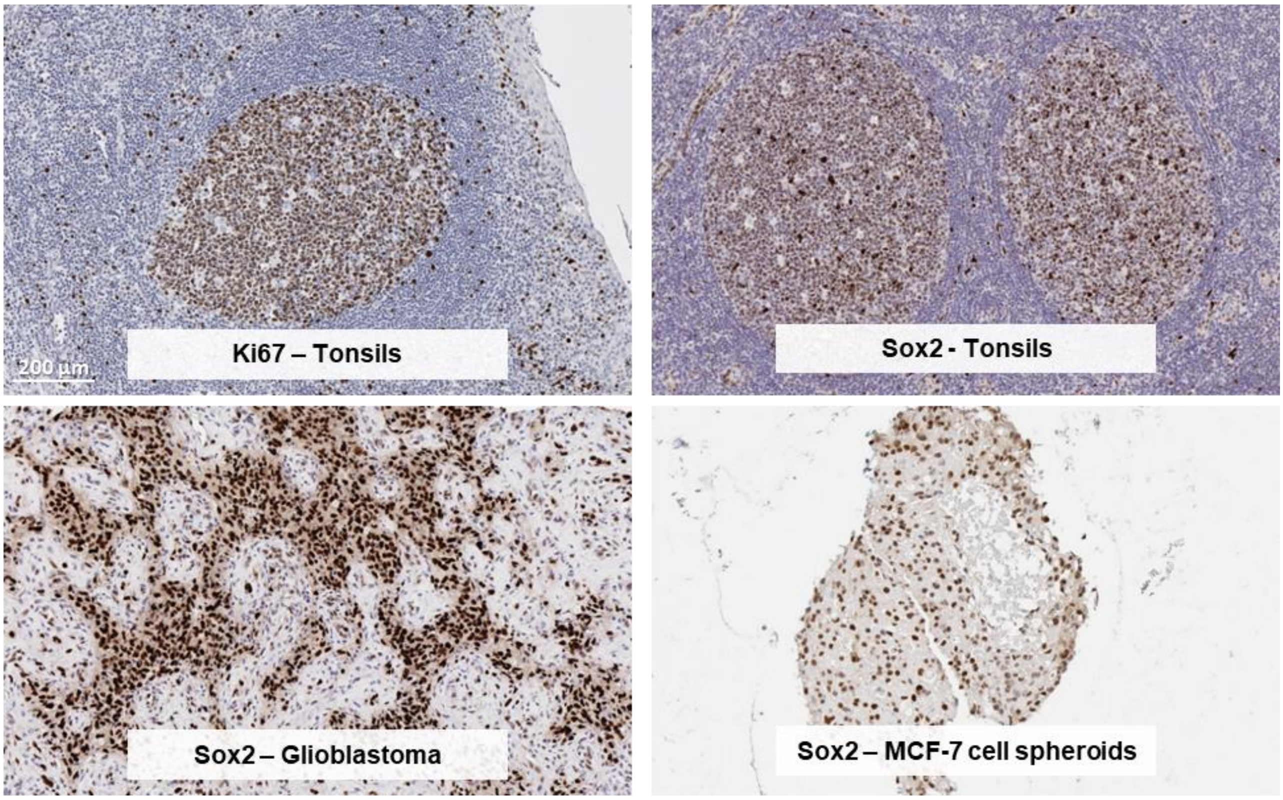

Figure S10: Representative images of control tissues used for Ki67 and Sox2 antibodies optimisation for immunohistochemistry. Scale bar represents 200 µm.
